# Projecting Uncertainty in Ecosystem Persistence Under Climate Change

**DOI:** 10.1111/gcb.70468

**Published:** 2025-09-02

**Authors:** Christina A. Buelow, Dominic A. Andradi‐Brown, Thomas A. Worthington, Maria F. Adame, Rod M. Connolly, Catherine E. Lovelock, Kerrylee Rogers, Jaramar Villarreal‐Rosas, Christopher J. Brown

**Affiliations:** ^1^ Coastal and Marine Research Centre, Australian Rivers Institute, School of Environment and Science Griffith University Gold Coast Queensland Australia; ^2^ Thriving Oceans Research Hub, School of Geosciences The University of Sydney Camperdown New South Wales Australia; ^3^ Ocean Conservation World Wildlife Fund Washington District of Columbia USA; ^4^ Department of Plant Sciences University of Cambridge Cambridge UK; ^5^ The University of Queensland School of Environment St. Lucia Queensland Australia; ^6^ School of Science University of Wollongong Wollongong New South Wales Australia; ^7^ Queensland University of Technology Centre for Environment and Society Brisbane City Queensland Australia; ^8^ Institute for Marine and Antarctic Studies University of Tasmania Taroona Tasmania Australia

**Keywords:** climate change, coastal, conservation, management, mangroves, modelling, projections, restoration

## Abstract

Global projections of ecosystem responses to increasing climatic and anthropogenic pressures are needed to inform adaptation planning. However, data of appropriate spatiotemporal resolution are often not available to parameterize complex environmental processes at the global scale. Modeling approaches that can project the probability of ecosystem persistence when parameter uncertainty is high may offer a way forward. In particular, the conservation of coastal ecosystems with complex dynamics, like mangrove forests, may benefit from knowing where their future persistence is highly probable or, alternatively, cannot be reliably estimated without additional data of appropriate resolution. Here, we simulated network models to make probabilistic projections of the direction of net change in mangrove ecosystems worldwide under the SSP5‐8.5 climate emissions scenario by the years 2040–2060. Seaward net loss was the most probable outcome in 77% [37%–78%; 95% confidence interval (CI)] of mangrove forest units, while 30% [15%–59%; CI] were projected to experience landward net gain or stability. In more than 50% of forest units, projections were ambiguous and therefore unreliable, with a near equal probability of net loss or gain. Quantitative models parameterized with locally accurate data could resolve uncertainty in the future persistence of mangroves in places with unreliable probabilistic projections. Projections made under conservation scenarios also showed that, with action to manage or restore, the number of mangrove forest units likely to experience net gain or stability in the future could nearly double. Our approach to simulating ecosystem responses to climatic and anthropogenic pressures provides a clear indication of how certain (or uncertain) ecosystem persistence is and thus can inform conservation planning.

## Graphical abstract

Projecting ecosystem change is essential for informed decision‐making. However, projections typically rely on complex models that require extensive knowledge and data, making large‐scale projections difficult. We show how probabilistic forecasts from qualitative network models can use coarsely resolved data to identify where ecosystem persistence under climate change is very likely, and where high uncertainty remains. This approach can guide priorities for improved data collection and conservation planning.

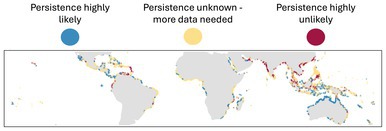

## 1 Introduction

As the pace of climate change and anthropogenic development increases (Folke et al. 2021), there is an urgent need to project the response of ecosystems to these pressures to inform management and conservation (Clark et al. 2001). However, projections are often inhibited by a lack of appropriate data. For example, projections could help managers avoid future collapse of river ecosystems, but data on key processes, such as relationships between fish survival and water flow regime, are often missing (Tonkin and Poff 2019). In addition, even data‐rich models that produce accurate and precise projections are not guaranteed to inform good decisions. This is partly because the reduction of parameter uncertainty required to optimize model skill can give managers a false sense of confidence that there is one unique “best‐fit” model—known as the “forecast trap” (Boettiger 2022). The risk of over‐confident decision‐making can be particularly high when making medium to long‐term projections of ecosystem change worldwide, in part due to the unavailability of global datasets of appropriate spatiotemporal resolution to parameterize complex processes underpinning ecosystem change. In other words, the resolution of available data and modeled processes is misaligned.

Network models can be used to project the probability of positive or negative ecosystem change when data are insufficient to parameterise complex processes and feedbacks (Marzloff et al. 2016; Melbourne‐Thomas et al. 2013). Network models encode a mechanistic understanding of how ecosystem components and external pressures interact, and interactions are qualified as either positive or negative. Given there is insufficient data for model parameterisation, simulation can be used to randomly parameterise the relative strength of ecosystem‐pressure interactions across a large sample of network models to reflect uncertainty (Melbourne‐Thomas et al. 2012). The suite of randomly parameterised network models can then be used to project the probability of the direction of net change of an ecosystem to increasing and potentially interacting pressures. Projecting the probability of the direction of net change of an ecosystem (i.e., net loss or gain), as opposed to making quantitative projections of the magnitude of change (e.g., change in ecosystem area) is prudent in data‐limited, high uncertainty situations, as overconfidence in fully quantitative projections could lead to management mistakes (Boettiger 2022; Raftery 2016). Projections with a near equal probability of decrease or increase in ecosystem extent will also signal to managers that, to make informed decisions, additional data is required to reduce uncertainty in the relative strengths of interactions in the network model. Furthermore, random parameterisation of the network model allows for novel combinations of ecosystem‐pressure interaction strengths to occur, so that projections are not constrained by what has occurred historically. Allowing for flexibility in dynamics is particularly important for ecosystems that may experience a reversal in historical trends in response to accelerating pressures, for example, mangroves responding to acceleration of sea level rise combined with coastal human pressures (Rogers 2021), and when making medium to long‐term projections of ecosystem change across regional or global scales. Probabilistic projections of ecosystem change under conservation and management scenarios derived from simulated network models could also help to identify where action is most likely to result in future persistence of an ecosystem and inform better management and conservation decisions (Nicholson et al. 2019).

Mangrove ecosystem dynamics are highly complex and therefore challenging to parameterise—particularly across large spatial scales. Quantitative models have projected high uncertainty in the magnitude of response of mangroves to future sea‐level rise; projected losses can range between 3% and 100% (Saintilan et al. 2023). This estimate of uncertainty is likely a dramatic under‐estimation, given that previous models have not considered the full range of human stressors impacting mangroves. Other important stressors include the frequency of intense storms, which affects erosion and drought, and damming of rivers, which affect sediment supply (Friess et al. 2019). These stressors interact through a dynamic geomorphic feedback process that governs whether mangrove forests accrete sediment and keep up with sea‐level rise or get drowned out (Rogers 2021). Mangrove extent can increase with sea‐level rise, as tidal inundation of coastal lands allows them to colonise new habitats further inland (Enwright et al. 2016). Sea‐level rise can cause loss of seaward mangroves, but mangroves can also prograde seaward as their complex root structures help to trap sediment, effectively raising the seabed and allowing them to expand their distribution into areas that would otherwise have been too deep (Woodroffe et al. 2016). These complexities make a challenging environment for decision‐makers, who need to seek climate‐smart conservation actions that are robust to high uncertainty (Hansen et al. 2010).

**FIGURE 1:**
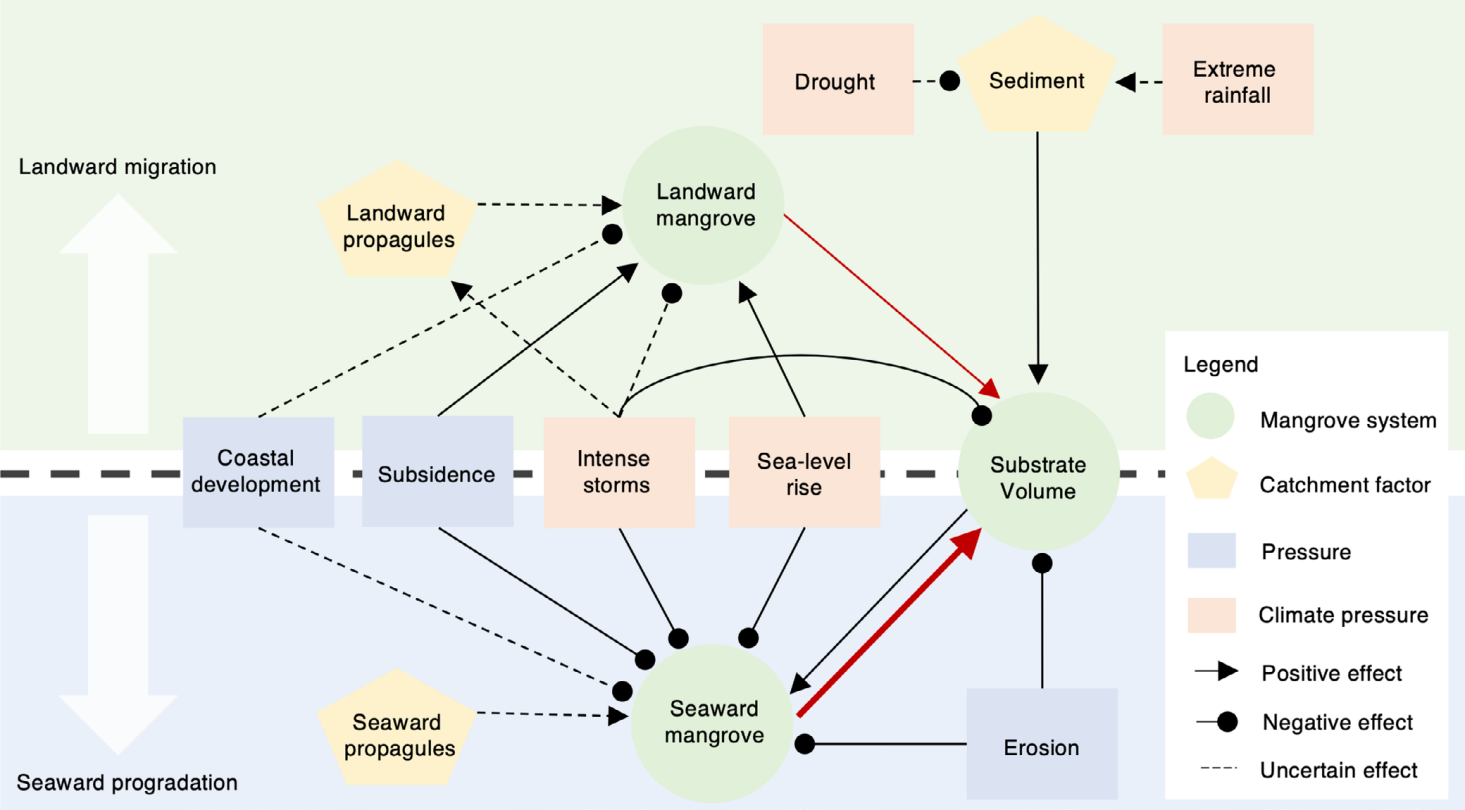
Network model representing mangrove landward migration or seaward progradation. All shapes in the network model represent nodes, and all lines represent edges (interactions between nodes). Edges with arrows (‐>) indicate a positive effect of one node on another, edges with circles (–•) indicate a negative effect, and dashed edges (‐‐‐) indicate an uncertain effect. The thickness of edges drawn in red indicate that the relative strength of the “Seaward mangrove” and “Substrate Volume” interaction is constrained to be greater than the “Landward mangrove” to “Substrate Volume” interaction. All nodes are self‐limiting (see methods for further explanation), but self‐limiting arrows are not shown for easier visualisation. Node definitions are provided in Table 1. The network model was simplified from an extended version (Figure S1) for which node and edge assumptions are defined in detail (Tables S1–S5).

Here, we developed a network model of landward and seaward mangrove extent change in response to increasing climatic and anthropogenic pressures (Figure 1). The model distinguishes between landward and seaward mangrove forest because their responses to pressures differ. We used the model to: (1) project the probability of mangrove net loss or gain/stability under scenarios of increasing pressures, (2) hindcast the probability of the direction of net change in mangrove extent across the globe and cross‐validate with historical observations derived from satellite remote sensing, (3) project the probability of the direction of net change in mangrove extent and compare to outcomes under management and restoration scenarios, and (4) identify where the direction of net change in mangrove extent is ambiguous and therefore not able to be projected without additional data to resolve parameter uncertainty. We aimed to demonstrate the utility of simulation‐based analysis of network models for making probabilistic projections that can inform management and conservation decisions at large spatial scales when data are limited and parameter uncertainty is high. Also, to improve model accuracy and ensure projection credibility (Meyer and Pebesma 2022), we fitted and validated the model with historical observations of mangrove responses to pressures that have played an important role in extent change over the recent past (1996–2020), but allowed for novel combinations of pressure interaction strengths with sea‐level rise, which is expected to increase substantially in the future (Friess et al. 2019).

## 2 Materials and Methods

### 2.1 Overview of the Simulation‐Based Modelling Approach

We used simulation‐based analysis of network models to hindcast and project the probability of mangrove ecosystem persistence in response to increasing anthropogenic and climatic pressures. Throughout the manuscript we use the term “hindcast” to refer to predictions by the model backwards in time and the term “projection” to refer to forward predictions in time that rely on assumptions about the future (i.e., projected pressures under shared socioeconomic pathway and climate emission scenarios) (Schoeman et al. 2023).

We chose to use this simulation‐based modeling approach instead of alternative approaches which are also able to project the probability of ecosystem change under future conditions (e.g., fuzzy cognitive mapping, Bayesian belief networks) because our focus was to understand the consequences of parameter uncertainty for making reliable projections to inform management and conservation decisions. Specifically, our approach was to randomly parameterize the relative strengths of interactions between ecosystem components and pressures. We then evaluated the consequences of this parameter uncertainty for the probability of predicted qualitative outcomes, that is, the direction of net change in an ecosystem (“gain/stability” vs. “loss”). An additional advantage of using network models was that they can include complex feedback loops between ecosystem components and pressures in the model structure. We designed our model to project mangrove ecosystem change at the catchment scale using globally available datasets on biophysical context and pressures, which resulted in a model structure that did not include multiple feedback loops (Figure 1). We anticipate, however, that future application of our modeling approach will be able to draw upon this important feature of network modeling.

Our workflow for modeling the probability of future net gain/stability or loss of mangrove ecosystems was:

1. Develop the network model structure (Figure 1).
2. Place constraints on the relative strength of interactions in the network model using prior knowledge of mangrove forest dynamics.
3. Simulate the probability of the direction of mangrove net change under increasing pressures.
4. Use global datasets to determine the biophysical contexts (e.g., tidal range and climate) and pressure levels (e.g., sea‐level rise and intense storms) in mangrove forest units globally (global datasets on pressures were both historical and medium‐term projections under emissions scenario SSP585).
5. Hindcast the probability of the direction of net change of each mangrove forest unit and use cross‐validation to (a) make fitted hindcasts (i.e., hindcasts where simulations are weighted by their likelihood), and (b) determine the optimal threshold for identifying when pressures are present or not.
6. Project the probability of the direction of net change of each mangrove forest unit using simulation likelihoods and pressure definition thresholds determined via cross‐validation.
7. Project the probability of the direction of net change of each mangrove forest unit under conservation and management scenarios.

These steps are described in detail in the subsections below. There are several sources of model uncertainty in our modeling approach, which we categorize and describe in Table 2.

### 2.2 Network Model Structure

We developed a network model to represent the interactions between factors influencing the landward and seaward extent of mangrove forests (Figure 1). The structure of our network model (Figure 1) was developed using the expert opinion of the authorship team and is a simplified version of a more complex model structure (Figure S1, Table S1) that captures additional processes influencing mangrove extent and was initially conceptualized from diagrams in Rogers (2021) and Rogers et al. (2023). The model structure was simplified so that: (1) landward and seaward mangrove ecosystem components include establishment space and established propagules, and (2) substrate volume and organic matter are a single ecosystem component. Our network model structure separated the response of mangrove forests to increasing pressures by the landward and seaward edge, as their response to pressures can be different. Our model also assumed that the boundary between seaward and landward mangroves is stable; therefore, a gain in seaward extent implies progradation, while a gain in landward extent implies migration landwards. Pressures included in the model structure were limited by whether there were global datasets available to indicate their presence in a mangrove forest in the past and in the future under climate change. We did not harness a key strength of qualitative network models, which is to evaluate the consequences of complex feedback loops between ecosystem components. Instead, we focused on developing a simulation approach that is simple enough to use available data, can be validated against observations, and can make probabilistic projections.

In the network model, each variable (ecosystem component or pressure) is a node and interactions (referred to as “edges”) are represented as lines between these nodes. All nodes in the network model were limited due to either density‐dependence (e.g., mangrove propagules cannot establish above a certain density threshold) or external factors not represented in the network model that will place limits on the extent to which they can increase (e.g., sedimentation at high rates cannot occur indefinitely as it is limited by tide heights, which are largely influenced by external factors). Node definitions are provided in Table 1. Edges are either positive (‐>) or negative (–•). A positive edge means that an increase in one node causes an increase in the other, while negative means a decrease. Prior information regarding edge occurrence probability and the relative strength of interactions can inform network model structure and parameter space. When probability of an edge interaction occurring is less than 100%, edges can be defined as uncertain (‐‐‐) and assigned a probability of occurrence based on expert understanding. The relative interaction strength of edges can also be constrained, either by ensuring that the relative strength of one edge is always higher than another (e.g., “Seaward Mangrove” and “Substrate Volume” is greater than the interaction strength between “Landward Mangrove” and “Substrate Volume”) (Figure 1) or by constraining the relative strength of one edge to be high or low by placing range limits on its parameter space (again, based on expert understanding).

**TABLE 1:** Network model node definitions. Nodes are variables in the model (Figure 1).

Category	Node	Definition
Mangrove ecosystem	Landward mangrove	Mangrove forest that can migrate landwards.
Mangrove ecosystem	Seaward mangrove	Mangrove forest that can prograde seaward.
Mangrove ecosystem	Substrate volume	The volume of substrate accumulating in seaward accommodation space (as defined by basement geology, high‐energy zone and highest astronomical tide (Rogers 2021)), including organic matter and sediment supplied from within the ecosystem or the upstream catchment or through marine tidal exchange.
Catchment factor	Landward propagules	Mangrove propagules that are available in the landward area.
Catchment factor	Seaward propagules	Mangrove propagules that are available in the seaward area.
Catchment factor	Sediment	Sediment from either the catchment or the marine environment.
Pressure	Erosion	Removal of substrate from the seaward shoreline.
Pressure	Coastal development	Development of human infrastructure near the coastline, such as roads and houses that directly reduces mangrove extent or potential for mangrove expansion.
Pressure	Subsidence from groundwater extraction or other processes, such as mining or oil/gas extraction	Human activity or local, natural processes causing downward movement of basement sediments (distinct from deep subsidence associated with isostatic or tectonic movement).
Climate pressure	Extreme high rainfall	Above average rainfall, high river flows and heightened sea levels.
Climate pressure	Drought	Below average rainfall, low river flows and dampened sea levels.
Climate pressure	Sea‐level rise	Sea‐level rise due to global warming.
Climate pressure	Intense storms	Impacts from intense tropical storms, including those from high wind speeds, wave and storm surges and high‐intensity rainfall.

**TABLE 2:** Sources of model uncertainty.

Category	Source of uncertainty	Description
Model structure	Probability of edge (interaction) occurrence	If an edge (i.e., interaction) between two nodes was uncertain to occur, the prior probability of occurrence was used to inform the proportion of matrix simulations the edge was present within the network model structure.
Model parameterisation	Parameter uncertainty	Simulation was used to randomly parameterise the relative strengths of interactions in the network model across a large sample of matrix simulations (*n* = 1000).
Model parameterisation	Pressure definition	Pressure presence was defined by thresholding the relative value of pressures across the globe. The relative value of pressures was estimated from globally available datasets, and the optimal threshold was identified from accuracy assessment of hindcasts.

**TABLE 3:** Model edge constraints or uncertainty representing different biophysical contexts. Edge constraints are either relative between two edges, as indicated by a greater than (>) or less than (<) arrows; binned using interaction strength ranges in square brackets [‐, ‐]; or are uncertain (‐‐) with probability of occurrence less than 100%. Interpretation: “{Sediment ‐> Substrate Volume} > {Sea‐level rise ‐* Seaward mangrove}” means that a constraint was enforced such that the positive sediment to substrate edge was greater in magnitude than the negative sea‐level rise to seaward mangrove extent edge. “{Sea‐level rise ‐* Seaward mangrove}; [−0.33, 0]” means that the negative sea‐level rise to seaward mangrove edge was constrained in the range of −0.33 to 0. “{Landward propagules ‐‐> Landward Mangrove}; probability = 10%” Means that the uncertain landward propagules ‐‐> landward mangrove edge was included with a probability of 10%. Edge constraint assumptions are provided in Table S5.

Variable	Setting	Edge/interaction strength constraint
Sediment supply	High	Sediment ‐> Substrate Volume > Sea‐level rise ‐* Seaward mangrove
	Low	Sediment ‐> Substrate Volume < Sea‐level rise ‐* Seaward mangrove
Tidal range	Macrotidal	Sea‐level rise ‐* Seaward mangrove; [−0.33, 0]
	Mesotidal	Sea‐level rise ‐* Seaward mangrove; [−0.34, −0.66]
	Microtidal	Sea‐level rise ‐* Seaward mangrove; [−1, −0.67]
Ecological connectivity	High	Land/seaward propagules ‐> Land/seaward mangrove; [0.67, 1]
	Medium	Land/seaward propagules ‐> Land/seaward mangrove; [0.34, 0.66]
	Low	Land/seaward propagules ‐> Land/seaward mangrove; [0.33, 0]
Future dams	Present	Sediment ‐> Substrate Volume < Sea‐level rise ‐* Seaward mangrove
	Absent	Sediment ‐> Substrate Volume > Sea‐level rise ‐* Seaward mangrove
Climate	Arid	Landward propagules ‐‐> Landward Mangrove; probability = 10%
	Humid	Landward propagules ‐‐> Landward Mangrove; probability = 50%
Human population density: coastal squeeze	High	Sea‐level rise ‐> Landward mangrove; [0.33, 0]
	Medium	Sea‐level rise ‐> Landward mangrove; [0.34, 0.66]
	Low/None	Sea‐level rise ‐> Landward mangrove; [0.67, 1]
Human population density: coastal development	High	Coastal development ‐‐* Landward mangrove; probability = 67%–100%
		Coastal development ‐‐* Seaward mangrove; probability = 0%–33%
	Medium	Coastal development ‐‐* Landward mangrove; probability = 34%–66%
		Coastal development ‐‐* Seaward mangrove; probability = 0%–33%
	Low	Coastal development ‐‐* Landward mangrove; probability = 0%–33%
		Coastal development ‐‐* Seaward mangrove; probability = 0%–33%
	None	Coastal development ‐‐* Landward mangrove; probability = 0%
		Coastal development ‐‐* Seaward mangrove; probability = 0%

### 2.3 Use Prior Understanding to Place Constraints on the Relative Strength of Interactions in the Network Model

We constrained the relative interaction strengths and probabilities of edge occurrence based on prior understanding of mangrove forest dynamics. These constraints were varied across different catchments to represent different biophysical contexts (Table 3). The use of prior information avoids unreasonable weights on extreme outcomes (Lemoine 2019). For example, the impact of coastal development on mangroves was assumed to be low in areas with low human population density (Table 3). Priors were informed by the literature and expert opinion (Table S5). Constraining interactions in network models with expert opinion is recommended when parameter uncertainty and system complexity are high because it prevents predictions that are inconsistent with current knowledge (Bode et al. 2017).

A long history of geomorphic research in mangroves provides a thorough empirical and theoretical basis for defining biophysical contexts and the constraints on edge weights (Rogers 2021). We developed the priors through extensive discussion with our coauthors who are experts in mangrove forest dynamics (TAW, MFA, RMC, CEL, KR, details Supporting Information; Tables S2–S5). The different biophysical contexts were: (1) high or low sediment supply, (2) macro‐, meso‐, or microtidal ranges, (3) high to low ecological connectivity, (4) high to low coastal squeeze, (5) high to low probability of coastal development negatively impacting mangroves, (6) presence of future dams, and (7) arid vs. humid climate (Table 3, Table S5).

### 2.4 Simulate the Probability of the Direction of Mangrove Net Change Under Increasing Pressures

We used the network model (Figure 1) to simulate the probability of net loss or gain/stability in the seaward and landward extent of mangroves in response to increasing pressures (Table 1) under different biophysical contexts (Table 3). Qualitative projections can be made via matrix algebra when network models are formulated as signed directed graphs (i.e., signed digraphs) which simply encode edges between nodes as positive or negative. Signed digraphs can be translated into a matrix of node interactions, where interaction coefficients are symbolic (i.e., represented with a + or ‐ sign), commonly referred to as a symbolic “community matrix” (Dambacher et al. 2002). To project the probability of loss and gain/stability in mangrove landward and seaward extent in response to increasing pressures (i.e., perturbations), we used simulation to randomly parameterize the edge weights and signs in a large sample (*n* = 1000) of community matrices that encoded a network model with a specific set of weights. Details and theoretical validation are provided in Melbourne‐Thomas et al. (2012), and we provide a summary here.

#### 2.4.1 Generate Randomized Community Matrices

We drew random numbers from uniform distributions to parameterize interaction coefficients of each community matrix in the sample. For unconstrained edges, we drew random numbers from a uniform distribution ranging [0, −1] if the sign of the interaction coefficient was negative, and [0, 1] if the sign of the interaction coefficient was positive (Melbourne‐Thomas et al. 2012). Where the relative strength of edge interactions within the model was constrained (i.e., sediment supply and dam biophysical scenarios; Table 3), constraints were defined using partial ordering and topological sorting that ensured interaction weights were parameterized to meet the constraint conditions (Ward et al. 2022). For edges with constraints on the range of weights, the random values were drawn from a uniform distribution with values in the given range (Table 3). Edges that were uncertain were first randomly sampled for inclusion using a Bernoulli distribution with a given probability (defaulted to 50%; see Table 3 for cases where uncertain edges had a probability of occurrence different from 50%). If included in a specific weights matrix, they were then assigned a random weight as above.

#### 2.4.2 Confirm Stability of Each Community Matrix

Second, for each random community matrix we checked stability. The community matrix is stable at equilibrium if all eigenvalues have a negative real part, meaning that a small change in a variable will not cause the ecosystem to collapse or shift to an alternate stable state. Stability is dependent upon the number and relative strength of negative and positive feedback loops in the network model, and instability occurs when either positive feedback loops dominate or when low‐level feedback (i.e., pairwise interactions) in the network is weak (Dambacher et al. 2003). We simulated random weights until we had 1000 stable matrices for different biophysical contexts (as defined by variables in Table 3). Matrix stability is a requirement for qualitative prediction of perturbation responses, and so unstable matrices were discarded before calculating the probability that seaward and landward mangroves will either decrease or increase/remain stable.

#### 2.4.3 Perturb Each Community Matrix According to Pressures

Each stable random community matrix can then be perturbed to predict the direction of net change in seaward and landward mangrove extent. The direct effect of a sustained increase or decrease in one variable (referred to as a “perturbation”) on other network variables can be predicted from the inverse of the community matrix if it is stable (Puccia and Levins 1985). Therefore, we positively perturbed (i.e., increased) pressure variables in the mangrove network models to make projections for the probability that seaward or landward mangrove extent will increase, decrease, or remain the same (i.e., stable). Throughout the manuscript, we group these outcomes into two binary categories for describing mangrove ecosystem net change: “loss” and “gain/stability.”

#### 2.4.4 Calculate Probability of Loss or Gain/Stability by Pooling Across Community Matrix Results

Finally, for each pressure scenario, the binary outcomes for direct of change for the mangrove extent variables were pooled across the 1000 community matrices to estimate the probability of gain/stability versus loss.

For our initial exploration of the projected probability of the future net change of mangrove ecosystems under increasing climatic and anthropogenic pressures, we made projections under scenarios where each individual pressure was increasing (i.e., drought, erosion, extreme rainfall, intense storms, subsidence, coastal development, and sea‐level rise) and where sea‐level rise was increasing in combination with all other individual pressures. To perform network model simulations, we used or adapted functions available in {QPress} (Melbourne‐Thomas et al. 2012) with R version 4.2.2 (R Core Team 2022).

### 2.5 Determine the Biophysical Contexts and Pressure Levels in Mangrove Forest Units Globally

The global distribution of mangroves was classified into 3983 units with the following geomorphic types: deltaic, estuarine, lagoonal, and open coast (Worthington et al. 2020), hereafter referred to as “mangrove forest units.” Each mangrove forest unit was characterised according to its biophysical context (Table 3) and the presence or absence of historical or future pressures using relevant global datasets (Table S6). Note that sea‐level rise was not included as a pressure in hindcasts because it is not a primary driver of mangrove extent change in the past 24 years (Friess et al. 2019), but it was included as a future pressure (see *Global projections and management/restoration scenarios* section below).

### 2.6 Global Hindcast Fitting and Cross‐Validation

We applied network models representing different biophysical contexts (Table 3) to the global distribution of mangrove forests (Bunting et al. 2022) to make probabilistic hindcasts of mangrove net loss or gain/stability. We used the hindcasts to estimate the likelihood of each random community matrix. The weighted average of the community matrices could then be used to create “fitted hindcasts” that is, hindcasts weighted by the probability of the direction of net change in mangrove extent. Hindcasts were measured against historical net change in mangrove extent derived from satellite remote sensing over a 24‐year period (1996–2020; Bunting et al. (2022)). Historical net change in mangrove extent in each mangrove forest unit was classified into binary outcomes of net gain/stability or net loss, allowing us to compare to the hindcast outcome.

We used cross‐validation to estimate error statistics and choose values of two hyper‐parameters that were necessary for making projections. Our hyper‐parameters were: (1) a threshold in the continuous pressure values that was used to define whether to perturb that pressure for the community matrix simulations at each mangrove unit (i.e., “pressure definition threshold”), and (2) when a probabilistic hindcast was classified as ambiguous (i.e., “ambiguity threshold”) meaning either net loss or gain/stability is probable. Pressure definition thresholds were grouped into five categories ranging from “very lenient” to “very strict” based on percentile or probability thresholds (e.g., any pressure above the 30th percentile was classified as present under the “very lenient” threshold—30th (very lenient), 40th (lenient), 50th (moderate), 60th (strict), and 70th (very strict); Table S6). Ambiguity thresholds ranged from 60% to 90% where, for example, a 60% ambiguity threshold meant that a hindcast with a probability of net loss or gain/stability less than 60% was “ambiguous.”

The coastal development pressure required special handling because a spatially explicit dataset on the presence of coastal infrastructure and development was not available. We therefore used human population density in the lower elevation coastal zone as a proxy for coastal development. Coastal development was considered present as a pressure in all units where human population density in the lower elevation coastal zone was greater than 0. We then modelled coastal development by allowing it to enter into the community matrix probabilistically. The probability it negatively impacting mangroves was dependent on whether population density was in “low,” “medium,” or “high” terciles (Table 3).

Our approach to hindcasting and cross‐validation was to randomly parameterize a large sample of matrices (*n* = 1000) for unique mangrove biophysical contexts identified globally (*n* = 74). These sets of randomly parameterized matrices were the basis for making hindcasts and projections in each mangrove forest unit, given its biophysical context, and did not change throughout hindcast and projection steps. We also distinguish between “un‐fitted” hindcasts and “fitted hindcasts.” Un‐fitted hindcasts are the probability of the direction of net change in mangrove forest extent estimated by solving the set of matrices (*n* = 1000) for the biophysical context and pressures present in a mangrove forest unit. The likelihood of each of the matrices in a set (*n* = 1000) was estimated via cross‐validation and used to make “fitted hindcasts,” that is, a weighted probability of the direction of net change in mangrove extent.

**FIGURE 2:**
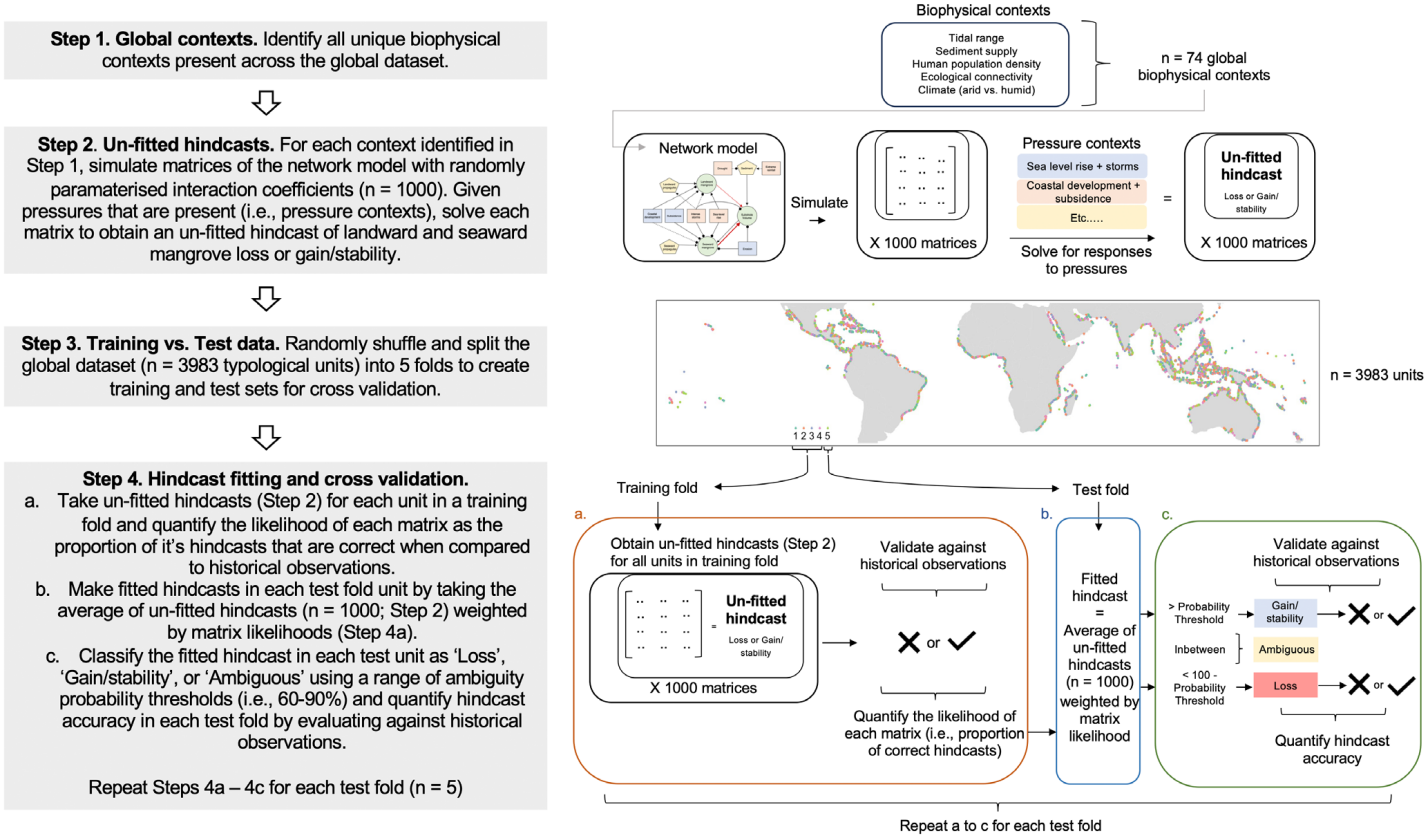
Model hindcast fitting and cross‐validation procedure. (Step 1) identify global biophysical contexts, (Step 2) make un‐fitted hindcasts given different biophysical and pressure contexts, (Step 3) Split global dataset into folds for training and testing, (Step 4) perform hindcast fitting and cross validation using training and test folds. Map lines delineate study areas and do not necessarily depict accepted national boundaries.

More specifically, our steps for making and cross‐validating fitted hindcasts of mangrove net change (Figure 2) were to: (1) identify all unique biophysical contexts (*n* = 74) present across the global dataset of mangrove forest units, (2) simulate randomly parameterized matrices (*n* = 1000) of the appropriately constrained network model (using the biophysical constraints from Table 3) for each biophysical context and solve each matrix for different combinations of pressures present across all units to make un‐fitted hindcasts of mangrove loss or gain/stability for different historical pressure contexts, (3) split the units (*n* = 3983) into five folds of training vs. test sets using simple random sampling (Wadoux et al. 2021), (4a) validate the un‐fitted hindcasts (*n* = 1000, step 2) against historical observations of net change for each unit in a training fold and quantify the likelihood of each matrix as the proportion of correct hindcasts in the training fold, (4b) fit the hindcasts by weighting the average of all un‐fitted hindcasts (step 2) by the likelihood of each matrix derived from the training folds (i.e., step 4a) for each unit in a test fold, (4c) classify the fitted hindcasts in the test fold as “gain/stability,” “ambiguous,” or “loss” using an ambiguity threshold (ranging from 60% to 90%), and quantify the accuracy of hindcasts in each test fold by evaluating against historical observations.

The above steps were then repeated for all combinations of ambiguity thresholds and pressure thresholds to create a grid of accuracy statistics. The optimized values for these two parameters was then simply the combination that had the highest cross‐validation accuracy. This approach is akin to the optimization of hyper‐parameters in other binary classification models, such as the number of trees in a boosted regression tree model. Accuracy metrics used were overall, user, and producer accuracy (Olofsson et al. 2014) (see steps in the following paragraph).

Accuracy metrics were calculated with the following equations:

(1) $$\text{Overall accuracy}=\frac{\mathrm{TP}+\mathrm{TN}}{\mathrm{TP}+\mathrm{TN}+\mathrm{FP}+\mathrm{FN}}$$

(2) $$\text{User accuracy}=\frac{\mathrm{TP}}{\mathrm{TP}+\mathrm{FP}}$$

(3) $$\text{Producer accuracy}=\frac{\mathrm{TP}}{\mathrm{TP}+\mathrm{FN}}$$

where TP = true positive, TN = true negative, FP = false positive, and FN = false negative. (Note that in classification problems, user accuracy is often called “precision” and producer accuracy is called “recall” [true positive rate]). Overall accuracy was measured across all hindcast classes (i.e., loss and gain/stability), while user and producer accuracy were measured for each individual hindcast class (i.e., loss or gain/stability). Ambiguous hindcasts were excluded from validation, as they cannot be evaluated against historical observations of net loss or gain/stability. Steps 1 to 4 were repeated for each pressure definition threshold, ranging from “very lenient” to “very strict.” The percentage of hindcasts that mis‐matched historical observations using optimal pressure definition and ambiguity thresholds was quantified for each marine ecoregion (Spalding et al. 2007).

To estimate uncertainty in our model's hindcast accuracy, we repeated the 5‐fold cross‐validation (*n* = 200, steps 3 and 4) using optimal thresholds to obtain resampled distributions for each accuracy metric (generating a total of 1000 test‐training pairs) and used the median and the 2.5th and 97.5th percentiles of each resampled distribution to quantify point estimates and 95% confidence intervals, respectively (Lyons et al. 2018). We propagated hindcast uncertainty through to the number of mangrove forest units projected to experience net loss or gain/stability by calculating 95% confidence intervals using error rates for each projection class with the following equations:

(4) $${\text{number}}_{i}\;95\%{\mathrm{CI}}_{\text{lower}}={\text{number}}_{i}-\left({\text{number}}_{i}*\left(1-\text{user}\;P_{5}\right)\right)$$

(5) $${\text{number}}_{i}\;95\%{\mathrm{CI}}_{\text{upper}}={\text{number}}_{i}+\left({\text{number}}_{i}*\left(1-\text{producer}\;P_{5}\right)\right)$$

where number_*i*_ is the number of mangrove forest units in projection class *i*, and ***P***_5_ is the 5th percentile of the user or producer accuracy for projection class *i*. Propagating error using Equations (4) and (5) allows for unsymmetrical error bounds, thereby enabling more useful interpretation of the uncertainty in projections for end‐users (Lyons et al. 2018, 2024; Murray et al. 2022).

### 2.7 Global Projections and Management/Restoration Scenarios

To make projections, we identified all unique biophysical contexts present in the future using projections of human population density by the year 2060 under shared socioeconomic pathway 5 (SSP5). We assumed that any historical coastal development acting as a barrier to landward migration of mangroves would be present in the future, and so therefore retained each unit's historical “coastal squeeze” status (Table 3) unless it was projected to increase in the future. Future pressure contexts were established using projected pressure data available in global datasets (medium term (2040–2060) projections; SSP5‐8.5; Table S6). The future presence of pressures in each mangrove forest unit was defined using the pressure definition threshold (ranging from “very lenient” to “very strict”; Table S6) that rendered the highest overall and user hindcast accuracy, except for future coastal development (whose presence was treated probabilistically (Table 3)) and sea‐level rise. Sea‐level rise was not included as a historical pressure in the hindcast cross‐validation and fitting procedure and therefore, to avoid under‐representing the impact of future sea‐level rise, we used the “very lenient” threshold to define its presence.

For future biophysical contexts that were present historically (*n* = 67), we solved randomly parameterized matrices (*n* = 1000) generated during hindcasting and used their likelihoods to make fitted projections under future pressures present in each mangrove forest unit. Alternatively, for future biophysical contexts without a historical analogue (*n* = 41), we generated new randomly parameterized matrices (*n* = 1000) and solved them to make un‐fitted projections under future pressures in each unit (i.e., step 2 of Figure 2). Projections were presented as the probability of net loss or gain/stability and further classified as either “net loss,” “net gain/stability,” or “ambiguous” according to the optimal ambiguity threshold identified from hindcast cross‐validation. Finally, Equations (4) and (5) were used to estimate 95% confidence intervals for the number of mangrove forest units in each projection class (i.e., net loss, net gain/stability, or ambiguous).

To make management and restoration scenario‐based projections, we positively perturbed additional nodes in the network model or altered interaction coefficients to correspond to the following management or restoration actions: (1) sediment addition (either through direct addition (Lopez‐Portillo et al. 2022) or by trapping with permeable fences (Winterwerp et al. 2020)) in units with riverine, tidal, or wave‐related sedimentary flows and erosion (i.e., deltaic, estuarine or lagoonal) (Balke and Friess 2016), (2) increase landward mangrove propagules via assisted dispersal (i.e., scattering of mangrove propagules) or enrichment planting (van Bijsterveldt et al. 2022), assuming propagules are a limiting factor in mangrove growth, (3) removal of coastal barriers that prevent landward migration of mangroves (Leo et al. 2019), or (4) improved ecological connectivity to facilitate propagule dispersal to areas biophysically suitable for growth (Pérez‐Ceballos et al. 2020), assuming a natural increase in propagule recruitment. Seaward mangrove planting is a restoration activity with historically low success (Lee et al. 2019; Samson and Rollon 2008) and so was not tested as a potential scenario.

For (1) sediment addition and (2) increase landward mangrove propagules via assisted dispersal scenarios, in addition to other future pressures present, we positively perturbed the “Substrate volume” and “Landward propagules” nodes, respectively. We therefore assume in (1) that any added or trapped sediment becomes additional substrate volume in the mangrove system. For (3) we simulated low coastal squeeze by setting the interaction coefficient between “Sea‐level rise” and “Landward mangrove” to be a randomly drawn number from a uniform distribution between 0.67 and 1 (Table 3), thereby assuming low constraints on the capacity for mangroves to migrate landward in response to sea‐level rise. For (4), we simulated improved ecological connectivity by setting the interaction coefficient between “Land/Seaward propagules” and “Land/Seaward mangrove” to “high ecological connectivity” (i.e., a randomly drawn number from a uniform distribution between 0.67 and 1; Table 3) and positively perturbed the “Landward propagule” and “Seaward propagule” nodes to simulate natural recruitment. To quantify the potential benefits of each management or restoration action, we estimated the number of mangrove forest units in each scenario‐based projection that: (1) switched from a baseline projection of net loss or ambiguity to become net gain/stability, and (2) switched from a baseline projection of net loss to a projection of > 50% probability of net gain/stability (referred to as “reduced certainty of loss”).

### 2.8 Sensitivity Analysis

We evaluated the sensitivity of projections to model assumptions by repeating scenario‐based simulations (*n* = 1000) with the following changes to the original network model (Figure 1): (1) extreme rainfall is certain to have a positive effect on sediment in the catchment, (2) drought is certain to have a negative effect on sediment in the catchment, (3) the negative effect of intense storms on seaward mangroves is uncertain, and (4) intense storms have a positive effect on substrate volume. For each of the four alternate models, we calculated the absolute deviation in the probability of gain/stability of landward and seaward mangroves from the probability estimated using the original network model (Figure 1) under each climatic and anthropogenic pressure scenario. We also used each of the four alternate models to project the direction of change in seaward and landward mangrove extent globally and identified where projected outcomes differed in comparison to projections made using the original network model (Figure 1).

## 3 Results

We have structured the results of our analyses according to our 4 sub‐aims as described in the introduction: (1) project the probability of mangrove net loss or gain/stability under scenarios of increasing pressures, (2) hindcast the probability of the direction of net change in mangrove extent across the globe and cross‐validate with historical observations derived from satellite remote sensing, (3) project the probability of the direction of net change in mangrove extent and compare it to outcomes under management and restoration scenarios, and (4) identify where the direction of net change in mangrove extent is ambiguous and therefore not able to be projected without additional data to resolve parameter uncertainty.

### 3.1 Projecting the Probability of Change in Mangrove Extent Under Increasing Climatic and Anthropogenic Pressure

**FIGURE 3:**
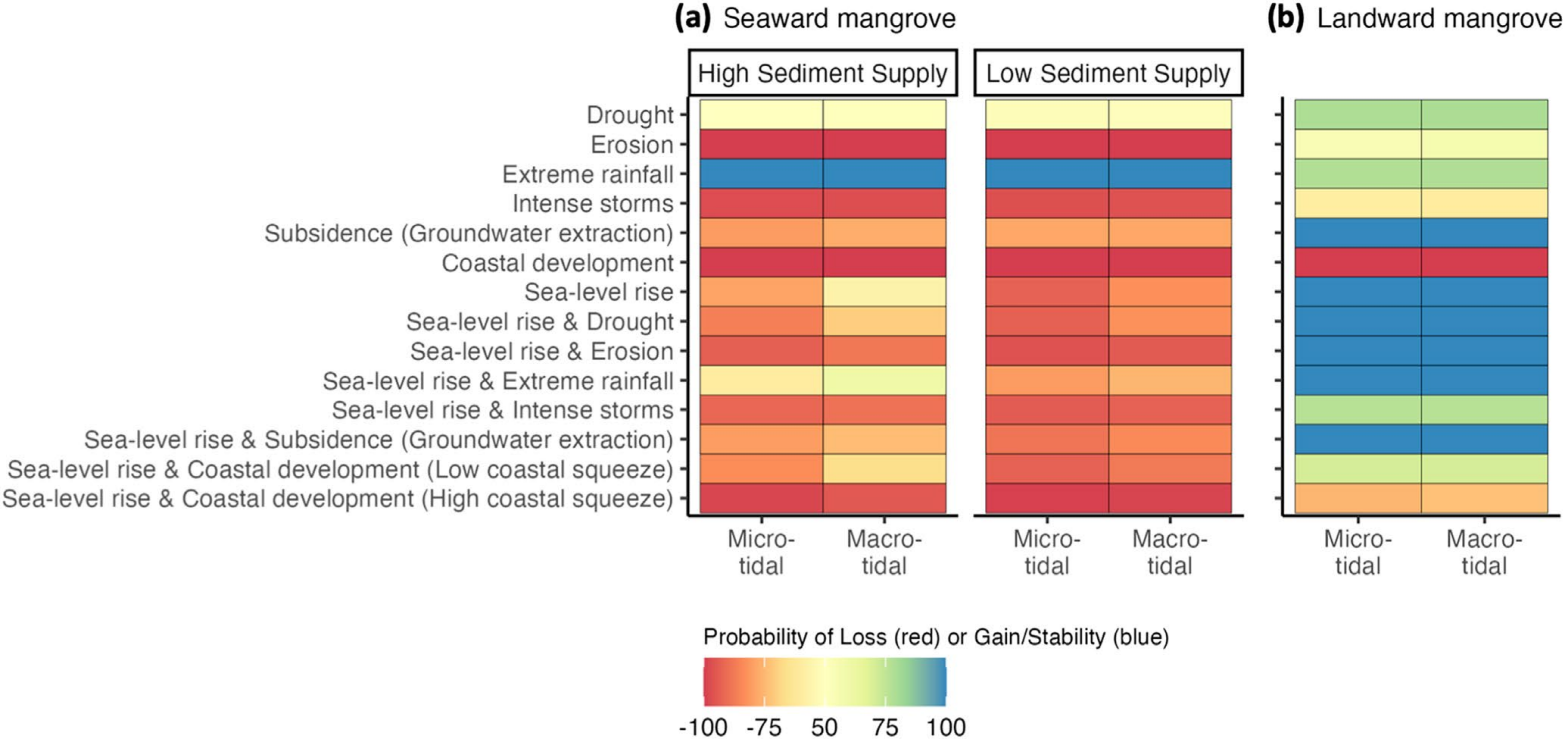
Probability of mangrove net loss or gain/stability under climatic and anthropogenic pressure scenarios. Probability of (a) seaward and (b) landward mangrove net loss or gain/stability as a proportion of simulated model outcomes (*n* = 1000). The probability of net loss or gain/stability can only be less than or greater than 50%, respectively, as outcomes are mutually exclusive.

Simulation‐based analysis of the network model (Figure 1) for different scenarios projected that seaward mangroves have a high probability of net loss in response to most climatic and anthropogenic pressures, except for increases in extreme high rainfall events (Figure 3a). In contrast, landward mangroves were projected to have a high probability of net gain under most pressure scenarios, except for those likely to experience loss from coastal development (Figure 3b). Generally, seaward mangroves were projected to have a lower probability of net loss when sediment supply was high. Seaward mangroves were also more likely to be lost in micro compared to macrotidal regions (Figure 3a). Landward mangroves were projected to have a higher probability of net loss with increasing sea‐level rise when coastal development limited space for landward migration (i.e., where “coastal squeeze” was high; Figure 3b).

### 3.2 Hindcasting the Probability of the Direction of Net Change in Mangrove Extent Across the Globe and Cross‐Validating With Historical Observations Derived From Satellite Remote Sensing

**FIGURE 4:**
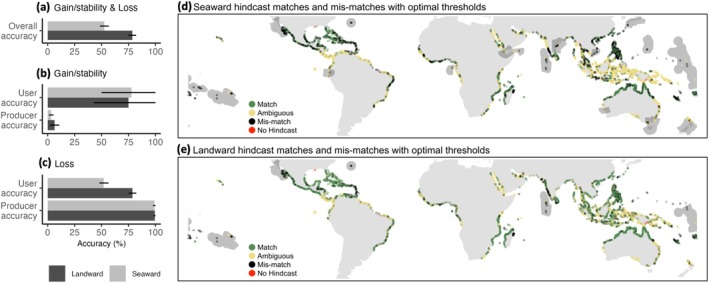
Hindcast accuracy. Hindcasts were evaluated against historical observations of net change in mangrove extent derived from satellite remote sensing over 24 years and were made using optimal pressure definition and ambiguity thresholds (i.e., “Strict” and “75%”, respectively; Figure S2). Hindcast accuracy was quantified via 5‐fold cross‐validation as: (a) overall accuracy of gain/stability and loss, (b) gain/stability user and producer accuracy, and (c) loss user and producer accuracy. Accuracy point estimates are median values, and error bars represent 95% confidence intervals, derived from resampled distributions of each accuracy metric. Maps display (d) seaward and (e) landward hindcast validation and discrepancies with historical observations. Red “No Hindcast” dots represent mangrove forest units where a hindcast was not possible due to the lack of a valid model (*n* = 7). Grey polygons represent marine ecoregion (Spalding et al. 2007) boundaries with greater than 80% discrepancy between hindcasts and historical observations. For a detailed description of datasets used for hindcasting see Table S6 or view interactively here: https://mangrove‐climate‐risk‐mapping.netlify.app. For more information on the hindcast fitting and cross‐validation procedure, see the methods and Figure 2. Map lines delineate study areas and do not necessarily depict accepted national boundaries.

Overall accuracy was based on evaluating hindcasts of loss and gain/stability together (excluding ambiguous hindcasts, which cannot be validated) and was lower for seaward (52%) than for landward mangroves (79%, Figure 4a). Producer accuracy (proportion of loss or gain/stability that was correctly hindcast) was consistently low for seaward and landward net gain/stability (< 7%, Figure 4b) but high for net loss (> 95%; Figure 4c). User accuracy (proportion of correct hindcasts of either loss or gain/stability) was 78% for hindcasts of seaward gain/stability, 75% for landward gain/stability, and 79% for landward loss (Figure 4b,c). In contrast, seaward loss user accuracy was low (50%, Figure 4c). Gain/stability user accuracy had higher uncertainty compared to other accuracy estimates (Figure 4a,b). Marine ecoregions (Spalding et al. 2007) with greater than 80% discrepancy between hindcasts and historical observations were similar for seaward and landward hindcasts (Figure 4d,e) and occurred in regions where the majority of mangrove forest units experienced both seaward and landward net gain/stability historically (see Figure S3 for percent of hindcast mismatches across all marine ecoregions).

The evaluation of hindcast accuracy indicates that, although the network model could not correctly predict all areas of historical net gain/stability (i.e., low producer accuracy; Figure 4b), hindcasts of net gain/stability and landward loss have high user accuracy. This means that most hindcasts of net gain/stability and landward loss were correct when compared to historical observations (Figure 4b,c). The user accuracy of seaward net loss was lower (Figure 4c), meaning that 50% of the hindcasts were in mangrove forest units with historical net gain/stability. The evaluation of hindcast accuracy offers insight into how the model could be improved if additional data on processes driving gains and losses become available.

### 3.3 Projecting the Probability of the Direction of Net Change in Mangrove Extent Globally

**FIGURE 5:**
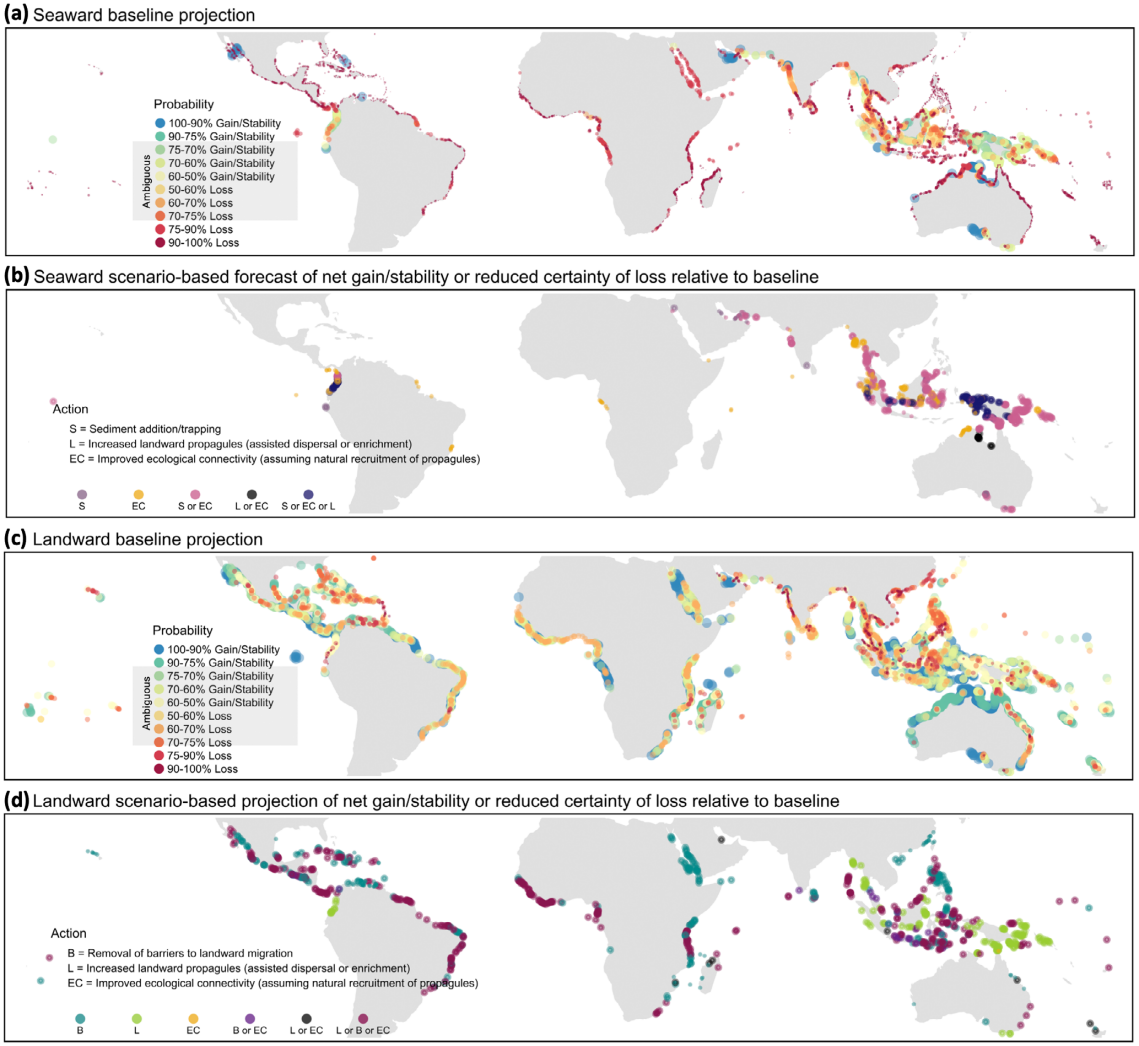
Probabilistic projections of the direction of change in mangrove extent globally. Baseline and scenario‐based projections of seaward (a, b) and landward (c, d) net change (view interactively here). For baseline projections (a, c), the size of each point corresponds to a gradient of high probability of net gain/stability (large points) to high probability of net loss (small points) for ease of visualisation where over‐plotting occurs. Projections were defined as “ambiguous” if the probability of net loss or gain/stability was less than 75% (Figure S2). Scenario‐based projections (b, d) show where management or restoration actions are projected to make net gain/stability certain (large points) or net loss less certain (small points) relative to the baseline (mangrove forest units with no change from the baseline are not shown). Management and restoration actions include sediment addition or trapping (S), increased ecological connectivity (assuming natural recruitment of propagules) (EC), removal of coastal barriers to landward migration (B), and increased landward mangrove propagules via assisted dispersal or enrichment planting (L) (each action was projected separately; see Methods for additional details). Mangrove forest units for which projections could not be made due to lack of a valid model (*n* = 7) are not displayed. See Figure S4 to visualise projections as mapped categories (i.e., net loss, gain/stability, or ambiguous). See Figure S7 for the pressure contexts associated with different categories of projection uncertainty. For a detailed description of datasets used for making projections, see Table S6. Map lines delineate study areas and do not necessarily depict accepted national boundaries.

We projected the probability of net loss or gain/stability in mangrove extent using pressures projected under emissions scenario SSP5‐8.5 for between the years 2040 and 2060 (Figure 5). We classified probabilistic projections into categories of “net loss” “net gain/stability”, or “ambiguous”. Ambiguous projections were defined when the probability of net loss or gain/stability was less than 75%, which was the optimal threshold identified in cross‐validation (Figure 5a,c; See Figure S4 for mapped categories of net change instead of the probabilities presented in Figure 5). We also made projections for management and restoration scenarios (Figure 5b,d) to identify where interventions could cause projections to change from: (1) a baseline projection of net loss or ambiguity to net gain/stability (i.e., > 75% probability of net gain/stability), or (2) a baseline projection of net loss to reduced certainty of loss (i.e., > 50% probability of net gain/stability).

Under the RCP 8.5 sea‐level rise scenario, 77% [37%–78%; 95% confidence interval (CI)] of mangrove forest units were projected to experience seaward net loss by 2060 (> 75% probability; Figure 5a), while only 13% [10%–14%; 95% CI] were projected to have landward net loss (Figure 5c). Landward net loss projections were prevalent along coastlines of the South Pacific Ocean, islands of the Lesser Antilles, the Persian Gulf, India, Bangladesh, Thailand, Vietnam, and southern Philippines (Figure 5c). Seaward net gain/stability was projected for 4% [2%–8%; 95% CI] of units (Figure 5a), while landward net gain/stability was projected in 30% [15%–59%; 95% CI] (Figure 5c). Seaward net gain/stability projections were concentrated in the Gulf of California, Persian Gulf, Arafura Sea, and southern Australia (Figure 5a), while landward net gain/stability was projected along coastlines of northern and southern Australia, central America, the Gulf of California and more intermittently along coastlines of west and east Africa, the Red Sea, Persian Gulf, south‐east Asia and New Caledonia (Figure 5c).

Under management and restoration scenarios we projected a switch from net loss or ambiguity to net gain/stability in either the landward or seaward edge of 26% of mangrove forest units, and reduced certainty of loss in an additional 6% of forest units (Figure 5b,d; see Methods “Global projections and management/restoration scenarios” for more detailed description of management and restoration interventions). More specifically, we projected seaward net gain/stability or reduced certainty of seaward loss in 10% and 4% of units, respectively, with action to increase or trap sediment, increase ecological connectivity (assuming natural recruitment of mangrove propagules), or increased landward propagule availability via assisted dispersal or enrichment planting (Figure 5b). The latter assumes that any increase in landward mangrove forest area from propagule establishment and growth also increases organic matter and substrate volume on the seaward edge, which can enhance the stability or increase the extent of seaward mangrove forest. Places where actions to manage or restore could result in seaward mangrove persistence were concentrated along coastlines of the South Pacific Ocean and southeast Asia (Figure 5b) where the baseline projection was ambiguous (Figure 5a). We also projected net gain/stability of landward mangroves or reduced certainty of loss in 18% and 2% of units, respectively, with action to remove coastal barriers to landward migration, increase ecological connectivity (assuming natural recruitment of mangrove propagules), or increase landward propagules via assisted dispersal or enrichment planting (Figure 5d). Compared to seaward mangroves, enhanced landward persistence of mangroves from management or restoration was more dispersed globally (Figure 5d).

### 3.4 Identifying Where the Direction of Net Change in Mangrove Extent Is Ambiguous

Projections were ambiguous in 19% [14%–60%; 95% CI] and 57% [27%–75%; 95% CI] of units for seaward and landward mangroves, respectively (Figure 5a,c). Ambiguous projections for both seaward and landward mangroves were concentrated in Papua New Guinea, the Solomon Islands, Myanmar, and Indonesia (Figure 5a,c). Projections were ambiguous on the seaward edge of coastlines along the South Pacific Ocean and Bangladesh (Figure 5a) and the landward edge of the northern Philippines, west and east Africa, Mexico, Brazil, and coastlines of the Caribbean Sea (Figure 5c).

### 3.5 Model Sensitivity

Network model projections were most sensitive to the assumption that drought and rainfall are uncertain to negatively and positively affect sediment availability, respectively. Assuming that drought is certain to have a negative effect on sediment availability resulted in a 50% change in the probability of seaward mangrove net gain/stability (Figure S5) and caused projected outcomes to change in 7% of mangrove forest units (Figure S6). Assuming that rainfall is certain to have a positive effect on sediment availability resulted in a 25% change in the probability of landward mangrove net gain/stability (Figure S5) and caused projected outcomes to change in 10% of mangrove forest units (Figure S6). Projections were moderately sensitive to the structural model assumption that intense storms negatively affect substrate volume; assuming a positive effect of intense storms caused the probability of mangrove net gain/stability to change by 20% (Figure S5) and projected outcomes to change in 7% of units (Figure S6). Projections were least sensitive to the assumption that intense storms are certain to have a negative effect on seaward mangroves, with less than 10% change in the probability of mangrove net gain/stability when the effect of intense storms was assumed to be uncertain (Figure S5), causing projected outcomes to change in 6% of units (Figure S6).

## 4 Discussion

Our findings of widespread seaward loss of mangroves, combined with gains at the landward edge, are consistent with other global models of mangrove extent change. Previous models suggest that coastal wetlands (including mangroves) in northern Australia, the Gulf of California, the Persian Gulf, the Gulf of Mexico, and East Africa are either unlikely to be lost to sea‐level rise (Lovelock et al. 2015) or may experience extent gains in the future (Saintilan et al. 2023; Schuerch et al. 2018). Generally, our projections also suggest net gain/stability or ambiguity (i.e., possible gains) in these regions. We also find that net loss of both landward and seaward mangroves is likely to be concentrated in Caribbean islands of the Lesser Antilles and Southeast Asia, aligning with previous models (Saintilan et al. 2023; Schuerch et al. 2018). Our projections also provide new information that is unavailable from other modeling approaches, including: (1) probabilistic projections of the direction of net change in extent for both the landward and seaward edge of mangroves, and (2) projections of the response of mangroves to pressures additional to sea‐level rise (e.g., intense storms, drought, and extreme rainfall).

Our probabilistic projections provide new insights into where there is uncertainty about the direction of mangrove forest responses to climate change. Uncertainty about whether forests will be lost or gained has important implications for mangrove conservation. In general, the direction of change was more certain for seaward than landward mangroves. This poses a challenge for conservation management, particularly actions that seek to protect and restore mangroves at the landward edge. For instance, many local human causes of degradation propagate from the landward edge of mangrove forests and therefore are places where conservation actions should also be taken. Locations with high uncertainty put in doubt the long‐term durability of this conservation. We suggest such locations are priorities for either climate‐smart actions that are robust to uncertainty (Hansen et al. 2010) (as described below), or for gathering additional information (e.g., finer‐scale, local data) to resolve parameter uncertainty and provide more precise projections. We also quantified other sources of projection uncertainty, namely model structure and prediction error; with propagation of the latter resulting in highly uncertain projections of seaward net loss and landward net gain/stability. Moving forward, greater model‐knowledge integration (Bradford et al. 2016) that focuses on including known geomorphic and hydrologic feedback processes that control seaward progradation into network model structure could help reduce these uncertainties. Overall, our modelling approach makes an important advance in quantifying and communicating uncertainty when undertaking the challenging task of projecting complex processes at the global‐scale with data of limited spatiotemporal resolution—which is crucial to moderate confidence in decision‐making and to avoid potentially suboptimal decisions (Boettiger 2022; Dietze 2017).

Our analysis of conservation scenarios highlighted significant opportunities for climate‐smart conservation actions to have a global impact on mangrove forest area. We found that conservation actions that increase ecological connectivity and/or remove barriers to landward migration allowed for a higher probability of persistence in the largest number of seaward and landward units, respectively. Increasing ecological connectivity could be achieved by restoring lost hydrological flow paths that would re‐connect mangroves (Pérez‐Ceballos et al. 2020), while the removal of barriers to landward migration (e.g., seawalls or levees) would require direct modification of coastal infrastructure (Leo et al. 2019). Both are climate‐smart actions because they facilitate natural processes that can help mangrove forests adapt to climate change (Purandare et al. 2024). While these actions have shown local success, our study emphasizes the necessity of enhancing their uptake globally. This can be achieved through the integration of climate‐smart planning into global best‐practices guides (e.g., Beeston et al. 2023), as well as directly into large‐scale programs that are seeking to upscale mangrove restoration, like the Mangrove Breakthrough (e.g., GMA 2024). Our conservation scenario‐based projections could be further refined by considering the socio‐economic and political feasibility of the proposed management and restoration actions, which would require regional or local information to assess (Budiharta et al. 2016). Paired with information on action feasibility, the probabilistic projections from our network model could be used to inform climate‐smart conservation plans (Buenafe et al. 2023).

Validation is a critically important step for communicating the credibility of projections to decision‐makers and ensuring that projections of conservation scenarios are providing robust recommendations to management (Bodner et al. 2021). We used historical observations of net change in mangrove extent over a 24‐year period to evaluate model hindcast accuracy. Our globally comprehensive hindcast validation differentiates our study from earlier models, which were only validated on spatially constrained areas, if at all. We found that overall, hindcast accuracy was higher for landward mangroves relative to seaward. User accuracy was high for hindcasts of net gain/stability and landward loss but low for seaward loss. From an end‐user's perspective, high user accuracy means that the model's projections of where gain/stability and landward loss will occur are reliable. Low user accuracy for seaward net loss could be due to time lags in the response of mangroves to inundation stress, or alternatively, that loss is obscured beneath the canopy of large shoreline mangroves and is therefore not observed with remote sensing techniques (Rogers and Saintilan 2021). Low producer accuracy for net gain/stability means that our model was unable to predict all areas of historical net gain/stability correctly. Such extensive validation allows transparency of the limitations of our network model and suggests priorities for future improvement.

The errors from validation point to two priorities for model refinement. First, the lower hindcast accuracy for seaward mangroves suggests integrating knowledge about progradation into the model is a priority for improving accuracy. Second, the marine ecoregions with the highest percent of hindcast discrepancies typically had experienced net gain/stability in both the landward and seaward forest edge. This suggests model accuracy could be improved through the inclusion of additional processes driving gains in mangrove extent, such as sediment delivery through land‐use change occurring upstream (Feller and Lovelock 2010; Lovelock et al. 2007). Additionally, historical observations of gain/stability in both the landward and seaward edge were less prevalent than observations of loss, meaning that fitting projections to historical observations may limit our capacity to adequately project gain/stability.

Our projections of mangrove persistence are likely to be conservative because of the caveats and limitations of our network model and modeling approach. The network model does not capture important local processes influencing the response of mangroves to sea‐level rise and other climatic and anthropogenic pressures, such as hydrodynamic energy and the shrink‐swell of organic matter; (see Rogers (2021) for a comprehensive overview of the topic). Our model is also unable to project poleward expansion of mangroves with increasing temperatures at latitudinal range limits (Saintilan et al. 2014), and we did not consider past or future land clearing as a driver of increased sediment in catchments, which could result in mangrove extent gains (Murray et al. 2022). We fitted our network model using historical observations of mangrove extent change; however, this could reduce the reliability of projections made under future pressures for which the historical observations cannot provide a robust analogue (Roberts et al. 2017). Furthermore, we made unfitted projections where future biophysical contexts had no historical equivalent (*n* = 41), which could reduce the accuracy of our propagated estimates of projection uncertainty. Although we evaluated the implications of parameter, model prediction, and model structural uncertainty for projected outcomes, more research is needed to consider uncertainty related to data inputs (i.e., pressure data), climate models, and emissions scenarios. Future research can also build upon our network model to overcome limitations and consider interactions with socio‐economic drivers of change (Hagger et al. 2022) and other coastal‐marine ecosystems that may be either negative or positive (Vozzo et al. 2023).

Lack of necessary or appropriate data is a major barrier to projecting for effective decision‐making. In an era of rapid environmental change, approaches that can leverage sparse data and effectively communicate any limitations and uncertainty that this imposes on our ability to make reliable projections will support better decisions. Here, we have combined network models with publicly available global datasets to make probabilistic projections of ecosystem net loss or gain in response to climatic and anthropogenic pressures. We have used cross‐validation and probabilistic thresholds that define projection ambiguity to make transparent the limits of our projections for informing decision‐making. By doing so, we show that projections of mangrove net loss or gain can be trustworthy in some locations and provide critical knowledge when imminent, science‐based decisions are required to maintain our planet's ecosystems.

## Author Contributions

**Christina A. Buelow:** conceptualization, data curation, formal analysis, funding acquisition, investigation, methodology, validation, visualization, writing – original draft, writing – review and editing. **Dominic A. Andradi‐Brown:** conceptualization, funding acquisition, investigation, methodology, resources, writing – review and editing. **Thomas A. Worthington:** data curation, investigation, methodology, writing – review and editing. **Maria F. Adame:** funding acquisition, investigation, methodology, writing – review and editing. **Rod M. Connolly:** funding acquisition, investigation, methodology, writing – review and editing. **Catherine E. Lovelock:** investigation, methodology, writing – review and editing. **Kerrylee Rogers:** investigation, methodology, writing – review and editing. **Jaramar Villarreal‐Rosas:** investigation, methodology, writing – review and editing. **Christopher J. Brown:** conceptualization, funding acquisition, investigation, methodology, project administration, supervision, writing – review and editing.

## Conflicts of Interest

Christopher J. Brown and Thomas A. Worthington are co‐leads of the Global Mangrove Alliance science working group, an organisation that promotes mangrove conservation.

## Supporting information


**Figure S1:** Extended mangrove network models with (A) catchment‐related factors, drought, erosion, and extreme rainfall, (B) sea‐level rise, (C) intense storms, (D) coastal development, and (E) subsidence.
**Figure S2:** Hindcast accuracy across all combinations of pressure definition and ambiguity thresholds and optimal “un‐fitted” hindcasts compared to “fitted.” Hindcast accuracy quantified via 5‐fold cross‐validation for (A) seaward and (B) landward mangroves using three metrics (producer, user, and overall accuracy) and a range of pressure definition and ambiguity thresholds. Comparison of “fitted” vs. “un‐fitted” hindcasts under the optimal pressure definition and ambiguity thresholds (i.e., “Strict” and 75%, respectively) for seaward (C, D) and landward (E, F) mangroves. The size of each point corresponds to a gradient of high probability of net gain/stability (large points) to high probability of net loss (small points). Grey dots in unfit hindcasts (C, D) represent units where a hindcast was not possible due to lack of a valid model. Map lines delineate study areas and do not necessarily depict accepted national boundaries.
**Figure S3:** Percent of hindcast mis‐matches by marine ecoregion for (A) seaward and (B) landward mangroves. Marine ecoregions with greater than 80% mis‐match are labelled by ecoregion name. Map lines delineate study areas and do not necessarily depict accepted national boundaries.
**Figure S4:** Baseline projections of (A) seaward and (B) landward net loss, gain/stability, or ambiguity. Probabilistic projections were classified as net loss, gain/stability, or ambiguity using the optimal ambiguity threshold (i.e., 75%) identified from hindcast cross‐validation. Map lines delineate study areas and do not necessarily depict accepted national boundaries.
**Figure S5:** Percent change in probability of mangrove gain/stability given different model assumptions from baseline model (Figure 1). (A) Intense storms were assumed to have a positive effect on substrate volume, (B) intense storms were assumed to have an uncertain negative effect on seaward mangroves, (C) drought was certain to have a negative effect on sediment, and (D) rain was certain to have a negative effect on sediment.
**Figure S6:** Change in projected outcome class (i.e., net loss, net gain/stability, ambiguous) given different model assumptions compared to the baseline model (Figure 1). Coloured points represent mangrove forest units where the outcome projected using the baseline model (Figure 1) is different from the outcome projected using models with the following alternative assumptions: (A) intense storms were assumed to have a positive effect on substrate volume, (B) intense storms were assumed to have an uncertain negative effect on seaward mangroves, (C) drought was certain to have a negative effect on sediment, and (D) rain was certain to have a negative effect on sediment. Colours and size of the points represent the four different alternative models. Map lines delineate study areas and do not necessarily depict accepted national boundaries.
**Figure S7:** Projection certainty and pressure context. Points represent individual mangrove forest units and their projected probability of net gain/stability on either the seaward or landward edge. Points are coloured by the mangrove forest's projection certainty classification, and different shapes represent the pressure context.
**Table S1:** Definition of network model nodes in Figure S1.
**Table S2:** Assumptions of the mangrove network model in Figure S1A.
**Table S3:** Assumptions for the network model with climate pressures sea‐level rise (Figure S1B) and intense storms (Figure S1C).
**Table S4:** Assumptions for the network model with anthropogenic pressures coastal development (Figure S1D) and subsidence (Figure S1E).
**Table S5:** Assumptions related to edge constraints or uncertainty representing different biophysical contexts (Table 3).
**Table S6:** Data sources for defining biophysical contexts and historical/future pressures in mangrove forest units (Worthington et al. 2020) representing the global distribution of mangroves (Bunting et al. 2022). View interactively here: https://mangrove‐climate‐risk‐mapping.netlify.app.

## Data Availability

The data that support the findings of this study are openly available in Zenodo at https://zenodo.org/doi/10.5281/zenodo.13668499.
