# Supplementary material for: Projecting Uncertainty in Ecosystem Persistence Under Climate Change
Source: Glob Chang Biol. 2025 Sep 2;31(9):e70468. doi: 10.1111/gcb.70468 (PMC12402971; doi:10.1111/gcb.70468)
Supplement: Supplementary file 1 — Figure S1: Extended mangrove network models with (A) catchment‐related factors, drought, erosion, and extreme rainfall, (B) sea‐level rise, (C) intense storms, (D) coastal development, and (E) subsidence. Figure S2: Hindcast accuracy across all combinations of pressure definition and ambiguity thresholds and optimal “un‐fitted” hindcasts compared to “fitted.” Hindcast accuracy quantified via 5‐fold cross‐validation for (A) seaward and (B) landward mangroves using three metrics (producer, user, and overall accuracy) and a range of pressure definition and ambiguity thresholds. Comparison of “fitted” vs. “un‐fitted” hindcasts under the optimal pressure definition and ambiguity thresholds (i.e., “Strict” and 75%, respectively) for seaward (C, D) and landward (E, F) mangroves. The size of each point corresponds to a gradient of high probability of net gain/stability (large points) to high probability of net loss (small points). Grey dots in unfit hindcasts (C, D) represent units where a hindcast was not possible due to lack of a valid model. Map lines delineate study areas and do not necessarily depict accepted national boundaries. Figure S3: Percent of hindcast mis‐matches by marine ecoregion for (A) seaward and (B) landward mangroves. Marine ecoregions with greater than 80% mis‐match are labelled by ecoregion name. Map lines delineate study areas and do not necessarily depict accepted national boundaries. Figure S4: Baseline projections of (A) seaward and (B) landward net loss, gain/stability, or ambiguity. Probabilistic projections were classified as net loss, gain/stability, or ambiguity using the optimal ambiguity threshold (i.e., 75%) identified from hindcast cross‐validation. Map lines delineate study areas and do not necessarily depict accepted national boundaries. Figure S5: Percent change in probability of mangrove gain/stability given different model assumptions from baseline model (Figure 1). (A) Intense storms were assumed to have a positive effect on substr [file GCB-31-e70468-s001.docx]

**Supporting information**


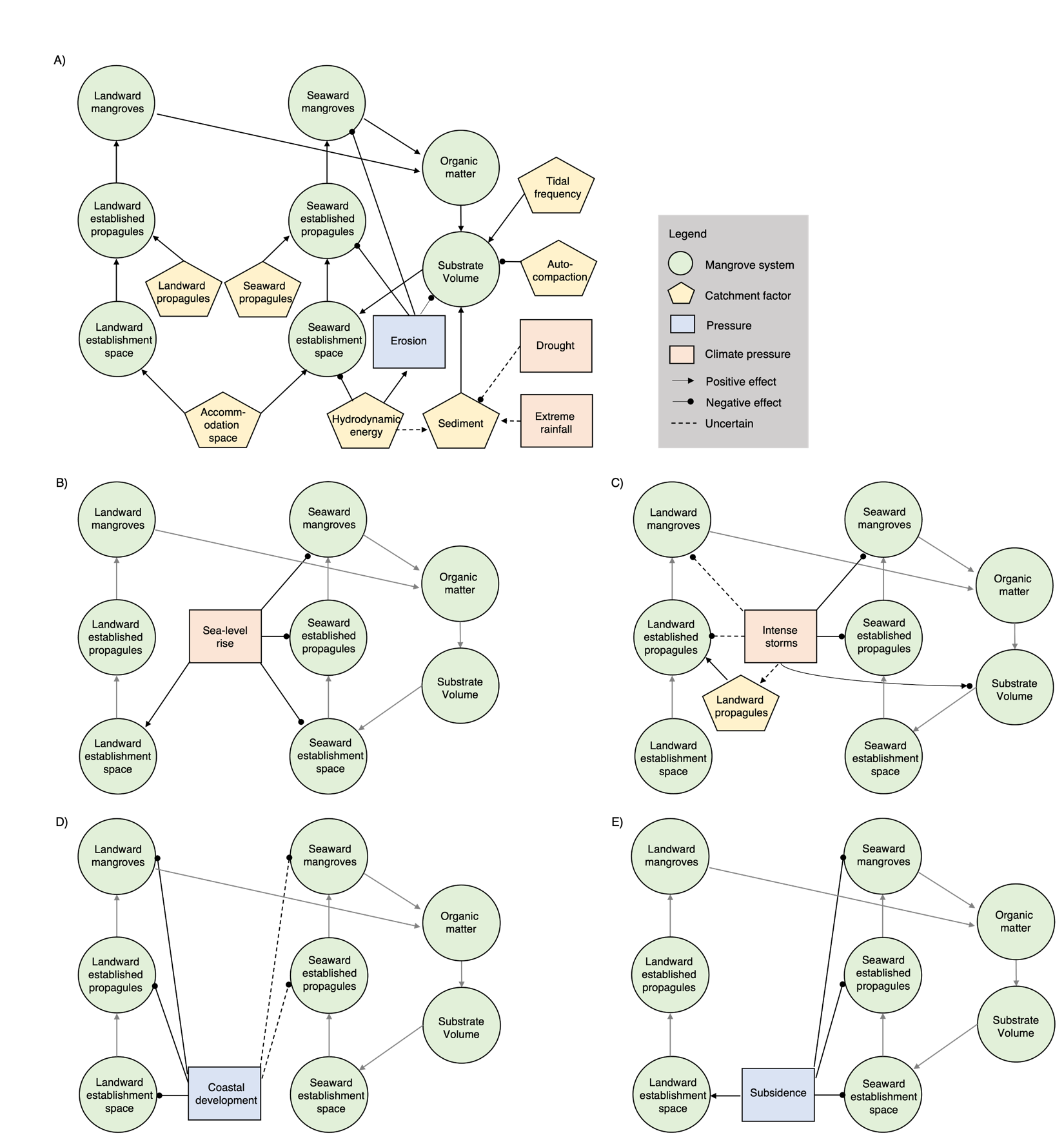


**Fig S1. Extended mangrove network models with A) catchment-related factors, drought, erosion, and extreme rainfall, B) sea-level rise, C) intense storms, D) coastal development, and E) subsidence.**

**
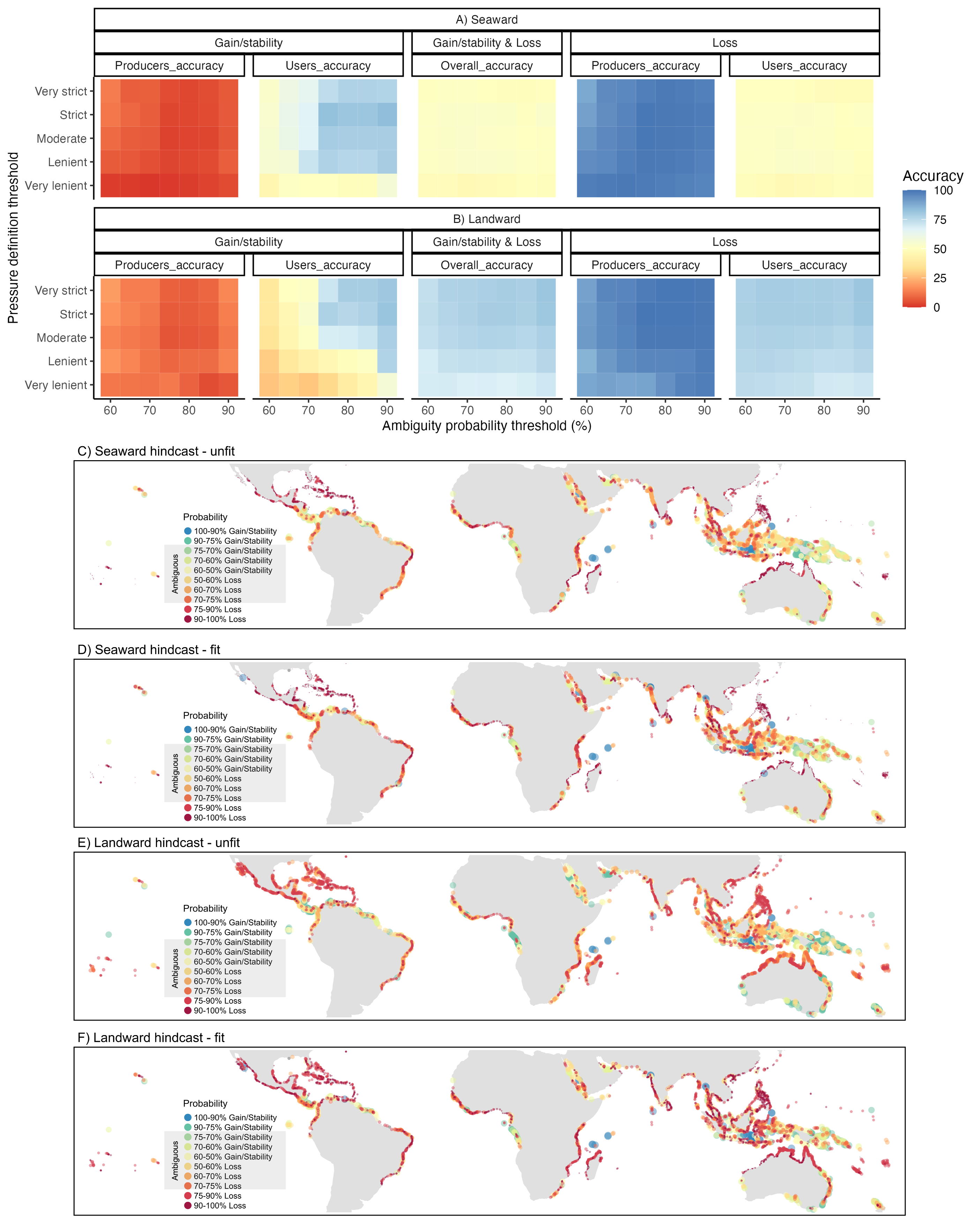
**

**Fig. S2. Hindcast accuracy across all combinations of pressure definition and ambiguity thresholds and optimal ‘un-fitted’ hindcasts compared to ‘fitted’.** Hindcast accuracy quantified via 5-fold cross-validation for A) seaward and B) landward mangroves using three metrics (producer, user, and overall accuracy) and a range of pressure definition and ambiguity thresholds. Comparison of ‘fitted’ vs ‘un-fitted’ hindcasts under the optimal pressure definition and ambiguity thresholds (i.e., ‘Strict’ and 75%, respectively) for seaward (C, D) and landward (E, F) mangroves. The size of each point corresponds to a gradient of high probability of net gain/stability (large points) to high probability of net loss (small points). Grey dots in unfit hindcasts (C, D) represent units where a hindcast was not possible due to lack of a valid model. Map lines delineate study areas and do not necessarily depict accepted national boundaries.

**
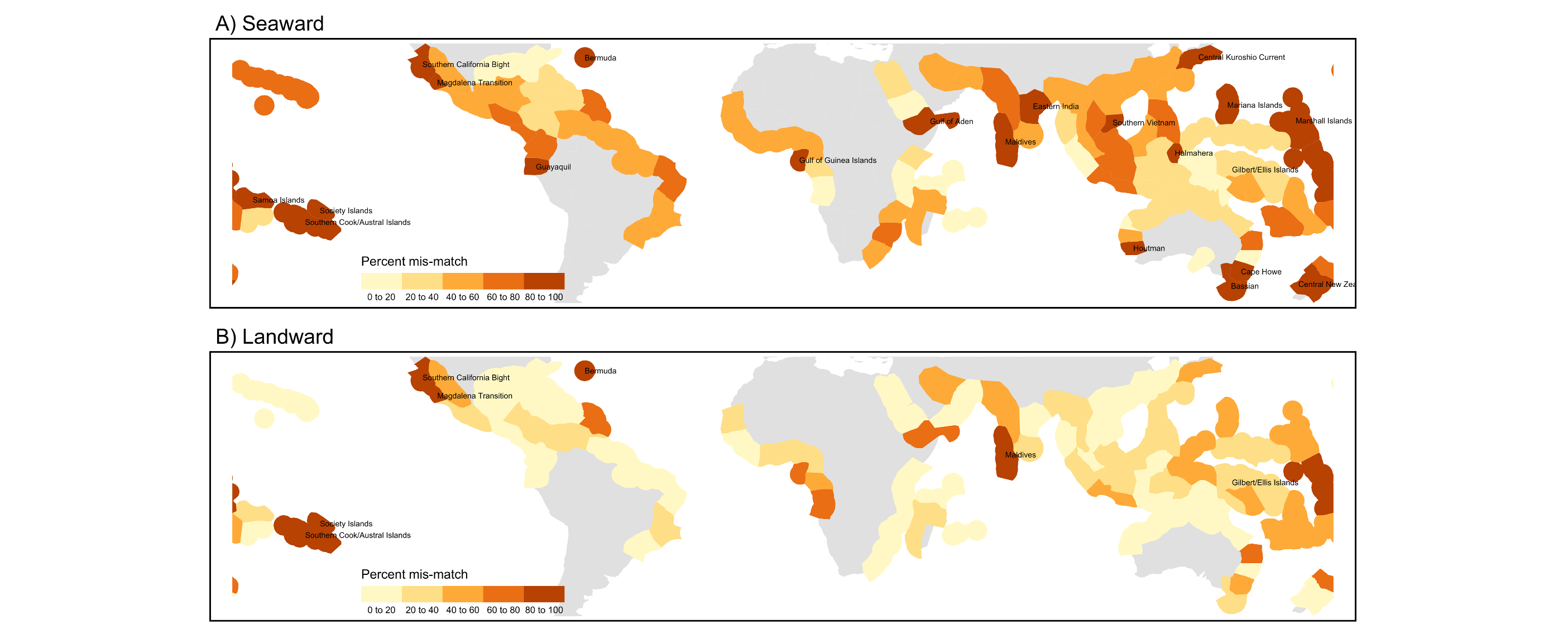
**

**Fig. S3. Percent of hindcast mis-matches by marine ecoregion for A) seaward and B) landward mangroves.** Marine ecoregions with greater than 80% mis-match are labelled by ecoregion name. Map lines delineate study areas and do not necessarily depict accepted national boundaries.

**Fig. S4. Baseline projections of A) seaward and B) landward net loss, gain/stability, or ambiguity**. Probabilistic projections were classified as net loss, gain/stability, or ambiguity using the optimal ambiguity threshold (i.e., 75%) identified from hindcast cross-validation. Map lines delineate study areas and do not necessarily depict accepted national boundaries.

**
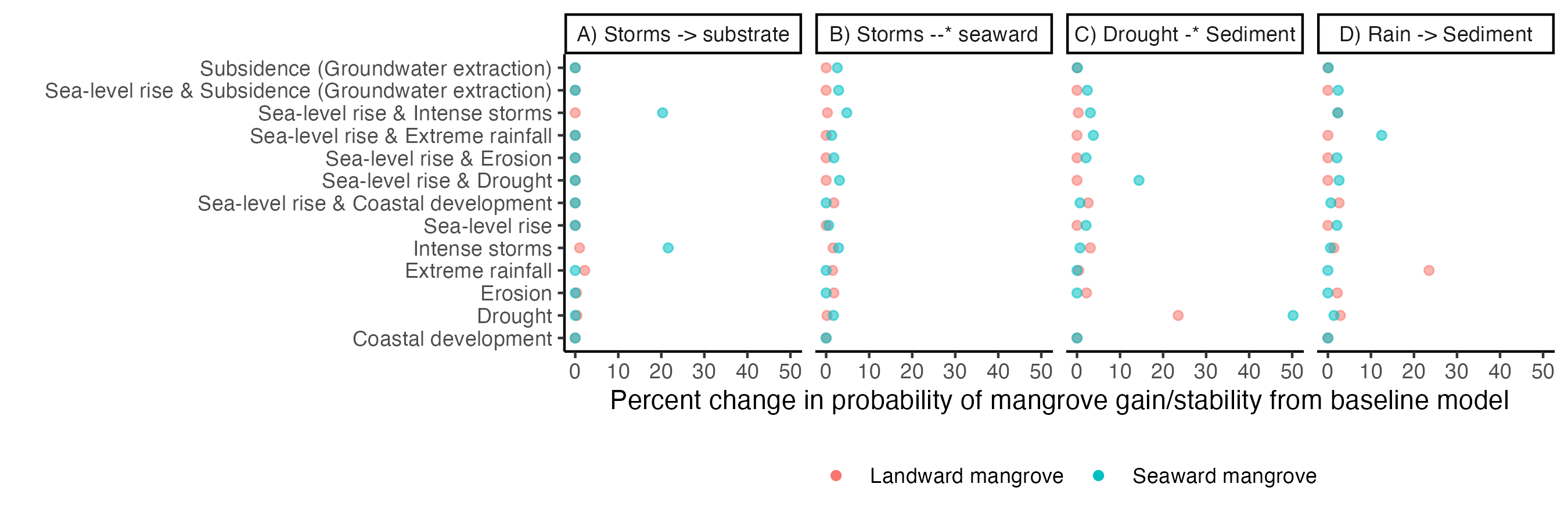
Fig. S5. Percent change in probability of mangrove gain/stability given different model assumptions from baseline model (Fig. 1).** A) Intense storms were assumed to have a positive effect on substrate volume, B) intense storms were assumed to have an uncertain negative effect on seaward mangroves, C) drought was certain to have a negative effect on sediment, and D) rain was certain to have a negative effect on sediment.

**Fig. S6. Change in projected outcome class (i.e., net loss, net gain/stability, ambiguous) given different model assumptions compared to the baseline model (Fig. 1).** Coloured points represent mangrove forest units where the outcome projected using the baseline model (Fig. 1) is different from the outcome projected using models with the following alternative assumptions: A) intense storms were assumed to have a positive effect on substrate volume, B) intense storms were assumed to have an uncertain negative effect on seaward mangroves, C) drought was certain to have a negative effect on sediment, and D) rain was certain to have a negative effect on sediment. Colours and size of the points represent the four different alternative models. Map lines delineate study areas and do not necessarily depict accepted national boundaries.


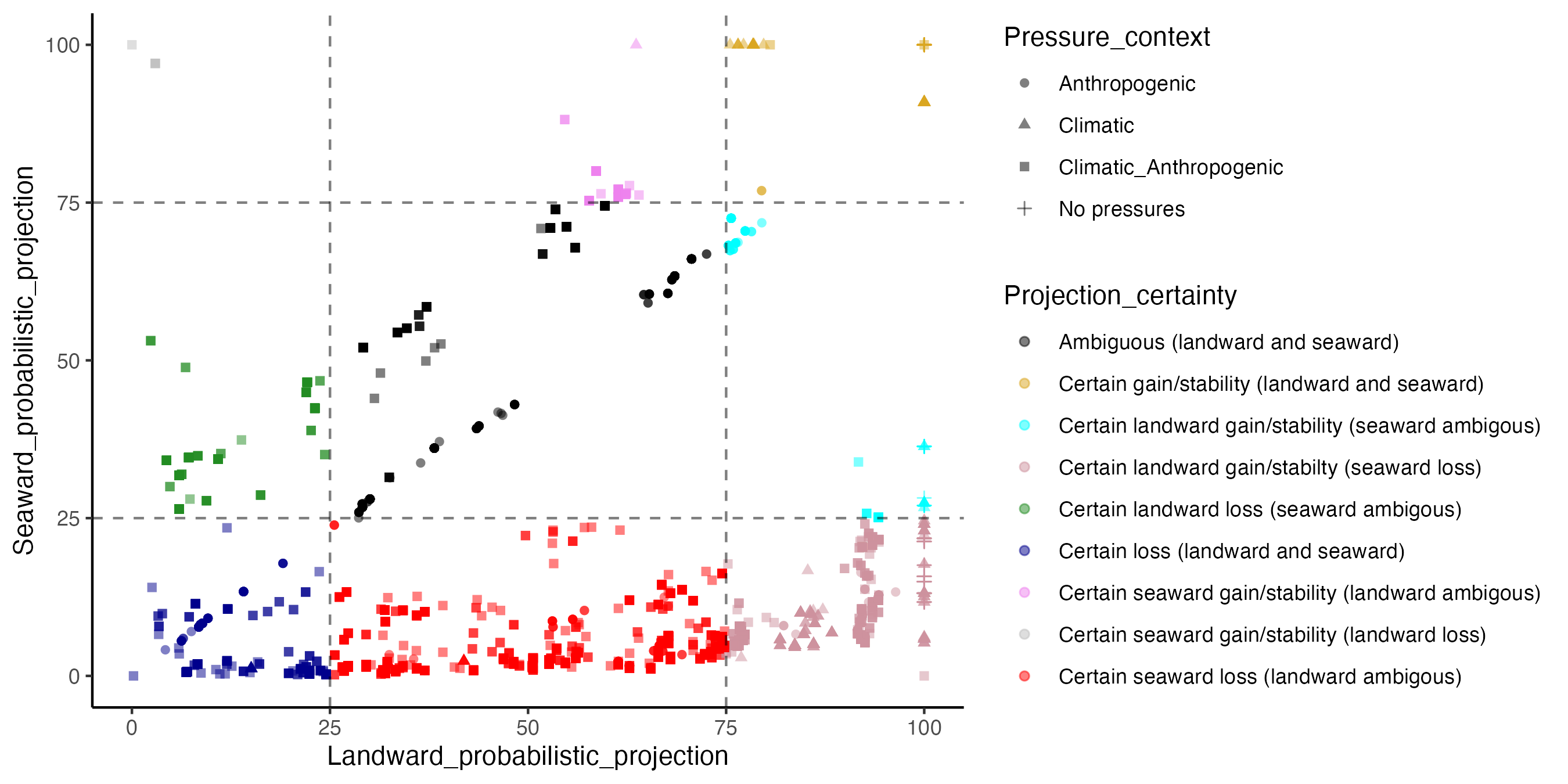


**Fig. S7. Projection certainty and pressure context.** Points represent individual mangrove forest units and their projected probability of net gain/stability on either the seaward or landward edge. Points are coloured by the mangrove forest’s projection certainty classification, and different shapes represent the pressure context.

**Table S1.** Definition of network model nodes in Fig S1.

| **Category** | **Node** | **Definition** |
| --- | --- | --- |
| Mangrove system | Landward and seaward establishment space | Space for mangrove propagules to establish and grow, towards either the land or sea. |
| Mangrove system | Landward and seaward establishing propagules | Area of establishing mangrove propagules, towards either the land or sea. |
| Mangrove system | Landward and seaward mangroves | Area of mangrove, towards either the land or sea. |
| Mangrove system | Organic matter | Organic matter from mangrove leaf litter and woody debris, or other plant or animal material (Rogers, 2021). |
| Mangrove system | Substrate volume | Volume of substrate that accumulates in the seaward establishment space from organic matter and sediment (supplied from either upstream river flows, marine tidal exchanges or produced in the mangroves). |
| Catchment factor | Landward and seaward propagules | Propagules that are available in the catchment, either dispersing landward or seaward. |
| Catchment factor | Accommodation space | Space where sediment and organic material can accumulate. |
| Catchment factor | Autocompaction | Decrease in substrate volume from either pressure of accumulating substrates/tidal water, or external decomposition and mineralisation of organic and carbon material (Rogers, 2021). |
| Catchment factor | Tidal frequency | Cycle of tidal inundation (i.e., semi-diurnal vs. diurnal) |
| Catchment factor | Sediment | Supply of sediment from the upstream catchment or marine environment. |
| Catchment factor | Hydrodynamic energy | Wind and wave energy that limits the amount of seaward space where substrate can accumulate, and mangrove propagules can germinate and grow. |
| Pressure | Erosion | Removal of sediment and substrate from seaward establishment space. |
| Pressure | Coastal development | Development of human infrastructure near the coastline, such as roads, houses, etc., that directly reduces mangrove extent. |
| Pressure | Subsidence from groundwater extraction or other processes, such as mining or oil/gas extraction | Human activity or local, natural processes causing downward movement of basement geology (distinct from deep subsidence associated with isostatic or tectonic movement). |
| Climate pressure | Extreme high rainfall | Above average rainfall, high river flows and heightened sea levels. |
| Climate pressure | Drought | Below average rainfall, low river flows and dampened sea levels. |
| Climate pressure | Sea-level rise | Sea-level rise due to global warming. |
| Climate pressure | Intense storms | Impacts from intense tropical storms, including those from high wind speeds, wave and storm surges and high intensity rainfall. |

**Table S2.** Assumptions of the mangrove network model in Fig S1A.

| **From** | **To** | **Edge/link direction** | **Assumption** |
| --- | --- | --- | --- |
| Landward establishment space | Landward established propagules | Positive | An increase in landward establishment space increases the potential for landward established mangrove propagules. |
| Landward established propagules | Landward mangroves | Positive | An increase in the number of established mangrove propagules increases the area of landward mangrove forest. |
| Landward mangroves | Organic matter | Positive | An increase in the amount of landward mangrove forest increases the amount of organic matter accumulated in the system. |
| Landward propagules | Landward established propagules | Positive | An increase in propagules available to establish landwards increases the amount of landward established propagules. |
| Organic matter | Substrate volume | Positive | An increase in organic matter increases substrate volume. |
| Substrate volume | Seaward establishment space | Positive | An increase in substrate volume increases the seaward space available for propagules to establish. |
| Seaward establishment space | Seaward established propagules | Positive | An increase in seaward establishment space increases the number of seaward established mangrove propagules. |
| Seaward establishing propagules | Seaward mangroves | Positive | An increase in the number of establishing seaward propagules increases the area of seaward mangrove forest. |
| Seaward mangroves | Organic matter | Positive | An increase in the amount of seaward mangrove forest increases the amount of organic matter accumulated in the system. |
| Seaward propagules | Seaward established propagules | Positive | An increase in propagules available to establish seawards increases the amount of seaward established propagules. |
| Accommodation space | Landward or seaward establishment space | Positive | An increase in accommodation space increases landward and seaward establishment space. |
| Tidal frequency | Substrate volume | Positive | Increased tidal frequency (e.g., a shift from diurnal tidal cycles to semi-diurnal tidal cycles) increases sediment accretion and therefore substrate volume (Belliard et al., 2023). |
| Autocompaction | Substrate volume | Negative | Increased autocompaction reduces substrate volume. |
| Sediment | Substrate volume | Positive | Increased sediment in the catchment increases substrate volume. |
| Hydrodynamic energy | Seaward establishment space | Negative | Increased hydrodynamic energy decreases seaward establishment space. |
| Hydrodynamic energy | Erosion | Positive | High hydrodynamic energy increases erosion. |
| Hydrodynamic energy | Sediment | Positive | High hydrodynamic energy can increase sediment availability. |
| Erosion | Substrate volume | Negative | Increased erosion decreases substrate volume. |
| Erosion | Seaward established propagules | Negative | Erosion can cause mortality of seaward established propagules. |
| Erosion | Seaward mangroves | Negative | Erosion can cause mortality of seaward mangroves. |
| Drought | Sediment | Negative | Reduced rainfall can decrease sediment available in the catchment. |
| Extreme rainfall | Sediment | Positive | Increased rainfall can increase sediment available in the catchment. |

**Table S3.** Assumptions for the network model with climate pressures sea-level rise (Fig S1B) and intense storms (Fig S1C).

| **From** | **To** | **Edge/link direction** | **Assumption** |
| --- | --- | --- | --- |
| Intense storms | Landward or seaward mangroves | Negative | Intense storms cause mortality of landward and seaward mangroves, but the strength of the link to landward mangroves is uncertain. |
| Intense storms | Seaward established propagules | Negative | Intense storms cause mortality of seaward established propagules. |
| Intense storms | Landward established propagules | Negative | Intense storms cause mortality of landward established propagules. |
| Intense storms | Landward propagules | Positive | Intense storms can deliver propagules landward via storm tides. |
| Intense storms | Substrate volume | Positive or negative | Intense storms cause can either decrease substrate volume (i.e., erosion; negative effect) or increase sediment accumulation (positive effect). |
| Sea-level rise | Landward establishment space | Positive | Sea-level rise directly increases landward establishment space. |
| Sea-level rise | Seaward establishment space, establishing propagules, and mangroves | Negative | Sea-level rise directly decreases seaward establishment space, establishing propagules, and mangroves. |

**Table S4.** Assumptions for the network model with anthropogenic pressures coastal development (Fig S1D) and subsidence (Fig S1E).

| **From** | **To** | **Edge/link direction** | **Assumption** |
| --- | --- | --- | --- |
| Coastal development | Landward establishment space, established propagules and mangroves | Negative | Increased coastal development directly decreases landward establishment space, establishing propagules and mangroves. |
| Coastal development | Seaward mangroves and established propagules | Negative | Increased coastal development directly decreases seaward mangroves and establishing propagules. |
| Subsidence | Landward establishment space | Positive | Subsidence directly increases landward establishment space. |
| Subsidence | Seaward establishment space, established propagules, and mangroves | Negative | Subsidence directly decreases seaward establishment space, establishing propagules, and mangroves. |

**Table S5.** Assumptions related to edge constraints or uncertainty representing different biophysical contexts (Table 3).

| **Biophysical setting** | **Assumption** | **References** |
| --- | --- | --- |
| Sediment supply | Supply of sediment to mangroves from the upstream catchment or marine environment can allow mangroves to persist under increasing sea-level rise. Therefore, when sediment supply is high, the relative strength of the positive effect of sediment on substrate volume is greater than the negative effect of sea-level rise on seaward mangrove. When sediment supply is low, the relative strength of the positive effect of sediment on substrate volume is lower than the negative effect of sea-level rise on seaward mangrove. | Sasmito, S. D., Murdiyarso, D., Friess, D. A., & Kurnianto, S. (2016). Can mangroves keep pace with contemporary sea level rise? A global data review. *Wetlands Ecology and Management*, *24*(2), 263–278. https://doi.org/10.1007/s11273-015-9466-7  Rogers, K. (2021). Accommodation space as a framework for assessing the response of mangroves to relative sea-level rise. *Singapore Journal of Tropical Geography*, *42*(2), 163–183. https://doi.org/10.1111/sjtg.12357 |
| Tidal range | The relative strength of the negative effect of sea-level rise on seaward mangrove is moderated by tidal range, because a higher tidal range helps deposit sediment, which in turn helps mangroves keep pace with sea-level rise (microtidal > mesotidal > macrotidal). | Sasmito, S. D., Murdiyarso, D., Friess, D. A., & Kurnianto, S. (2016). Can mangroves keep pace with contemporary sea level rise? A global data review. *Wetlands Ecology and Management*, *24*(2), 263–278. https://doi.org/10.1007/s11273-015-9466-7  Rogers, K. (2021). Accommodation space as a framework for assessing the response of mangroves to relative sea-level rise. *Singapore Journal of Tropical Geography*, *42*(2), 163–183. https://doi.org/10.1111/sjtg.12357 |
| Ecological connectivity | The relative strength of the positive effect of landward or seaward propagules on landward or seaward mangrove forest, respectively, is moderated by average distance between extant and lost mangrove forest – areas closer to propagule sources are more likely to recruit propagules (low distance > medium distance > high distance). | Van der Stocken, T., Wee, A. K. S., De Ryck, D. J. R., Vanschoenwinkel, B., Friess, D. A., Dahdouh-Guebas, F., Simard, M., Koedam, N., & Webb, E. L. (2019). A general framework for propagule dispersal in mangroves. *Biological Reviews*, *94*(4), 1547–1575. https://doi.org/10.1111/brv.12514 |
| Dams | Dams prevent sediment from reaching the coastline. Therefore, when dams are present, the relative strength of the positive effect of sediment on substrate volume is lower than the negative effect of sea-level rise on seaward mangrove. When dams are not present, the relative strength of the positive effect of sediment on substrate volume is greater than the negative effect of sea-level rise on seaward mangrove. | Syvitski, J. P. M., Vörösmarty, C. J., Kettner, A. J., & Green, P. (2005). Impact of Humans on the Flux of Terrestrial Sediment to the Global Coastal Ocean. *Science*, *308*. <https://www.science.org>  Ezcurra, E., Barrios, E., Ezcurra, P., Ezcurra, A., Vanderplank, S., Vidal, O., Villanueva-Almanza, L., & Aburto-Oropeza, O. (2019). A natural experiment reveals the impact of hydroelectric dams on the estuaries of tropical rivers. *Science Advances*, *5*, 9875–9888. https://www.science.org |
| Climate | The probability of landward propagules establishing and increasing landward mangrove forest area is higher in humid climates compared to arid climates because of highly variable and extreme temperatures and salinities. | Ferreira, A. C., de Lacerda, L. D., Rodrigues, J. V. M., & Bezerra, L. E. A. (2023). New contributions to mangrove rehabilitation/restoration protocols and practices. *Wetlands Ecology and Management*, *31*(1), 89–114. https://doi.org/10.1007/s11273-022-09903-2  Gorman, D., Vanderklift, M. A., & Lafratta, A. (2022). Quantitative Analysis of Methodological and Environmental Influences on Survival of Planted Mangroves in Restoration and Afforestation. *Forests*, *13*(3). https://doi.org/10.3390/f13030404 |
| Human population density | Human population density in the lower elevation coastal zone of the catchment surrounding each mangrove forest determines:  1) ‘Coastal squeeze’, i.e., the relative strength of the positive effect of sea-level rise on landward mangrove (zero or low population density > medium population density > high population density).  2) The probability of coastal development occurring and negatively impacting landward mangroves (zero population density = 0 probability of coastal development, low population density = 0-33% probability of coastal development, medium population density = 34-66% probability of coastal development, high population density = 67-100% probability of coastal development) and seaward mangroves (zero population density = 0 probability of coastal development; low, medium or high population density = 0-33% probability of coastal development). We therefore assume that the probability of coastal development negatively impacting seaward mangroves is always low. | Rogers, K., Mogensen, L. A., Davies, P., Kelleway, J., Saintilan, N., & Withycombe, G. (2019). Impacts and adaptation options for estuarine vegetation in a large city. *Landscape and Urban Planning*, *182*, 1–11. https://doi.org/10.1016/j.landurbplan.2018.09.022 |

**Table S6. Data sources for defining biophysical contexts and historical/future pressures in mangrove forest units** (Worthington et al., 2020) **representing the global distribution of mangroves** (Bunting et al., 2022)**.** View interactively here: [https://mangrove-climate-risk-mapping.netlify.app](https://mangrove-climate-risk-mapping.netlify.app/).

| **Category** | **Node** | **Description** | **Calculation** | **Data source** | **Definition in network models** (pressure definition thresholds categorized as very lenient, lenient, moderate, strict, or very strict) |
| --- | --- | --- | --- | --- | --- |
| Historical pressure | Antecedent sea-level rise | Historical sea-level rise impacting mangroves. | Regional mean sea level trends (mm/yr, 1993-2015) were estimated for each mangrove forest unit by obtaining the raster pixel value (resolution of 1/4°) closest to the centroid of each unit. Extreme values were truncated at > \|5\|mm yr-1 (Worthington et al. in prep). Units with sea-level rise values above a percentile threshold were considered at risk from future sea-level rise impacts. | Regional mean sea level trends (1993-2015); [https://climate.esa.int/en/projections/sea-level/data/](https://climate.esa.int/en/projects/sea-level/data/)    <https://catalogue.ceda.ac.uk/uuid/3ac333b828b54e3495c7749f5bce2fe3> | Classified as impacted by sea-level rise if above a percentile threshold: 30^th^ (very lenient), 40^th^ (lenient), 50^th^ (moderate), 60^th^ (strict), and 70^th^ (very strict). |
| Future pressure | Sea-level rise | Future sea-level rise impacting mangroves. | Medium term (2041-2060) sea-level rise (m) relative to baseline (1995-2014) conditions for each mangrove forest unit was estimated from an ensemble mean of CMIP6 projections under scenario SSP5-8.5 (raster with 1° resolution) ​(Gutierrez et al., 2021)​. The raster pixel value closest to the centroid of each mangrove forest unit was obtained. Units with future sea-level rise values above a percentile threshold were considered at risk from future sea-level rise impacts. | IPCC WGI Interactive Atlas; https://interactive-atlas.ipcc.ch | Classified as impacted by future sea-level rise if above a percentile threshold: 30^th^ (very lenient), 40^th^ (lenient), 50^th^ (moderate), 60^th^ (strict), and 70^th^ (very strict). |
| Historical pressure | Coastal development and squeeze | Coastal development preventing landward migration of mangroves. | Human population density in the lower elevation coastal zone (10m elevation above mean sea level ​(CIESIN, 2021)​) for the year 2000 (raster of ~1km resolution, adjusted to match UN country totals) ​(CIESIN, 2011)​ was used as a proxy for coastal infrastructure that reduces space available for mangroves to migrate landward. Total population size within the lower elevation coastal zone (LECZ) of each mangrove forest unit’s catchment was calculated and divided by the total area of the LECZ in that catchment to obtain a density estimate. For units without a mangrove catchment, a 50km buffer was used to calculate population density in the LECZ nearby the unit. If there was no LECZ within the 50km buffer, the unit was assigned the maximum population density estimate from across all units to indicate there is no space available for mangroves to migrate landward. Units were then classified as ‘high’, ‘medium’ or ‘low’ coastal squeeze using terciles on logged values above 0, or as ‘none’ if population density was 0. | Human population density in the lower-elevation coastal zone (within 10m of mean sea-level); https://sedac.ciesin.columbia.edu/data/set/grump-v1-population-count/data-download | Classified as high, medium or low coastal squeeze using terciles, or as ‘none’ if population density was 0. Coastal development was defined as present if population density was not ‘none’. |
| Future pressure | Coastal development and squeeze | Future coastal development preventing landward migration of mangroves. | Projected human population size by the year 2060 (SSP5; raster of 1km resolution) ​(Merkens et al., 2016)​ located within 10m elevation above mean sea level (lower elevation coastal zone; ​(CIESIN, 2021)​) was used as a proxy for the future development of coastal infrastructure that reduces space available for mangroves to migrate landward with climate change. Total population size within the lower elevation coastal zone (LECZ) of each mangrove forest unit’s catchment was calculated and divided by the total area of the LECZ in that catchment to obtain a density estimate. For units without a mangrove catchment, a 50km buffer was used to calculate population density in the LECZ nearby the unit. If there was no lower elevation coastal zone within the 50km buffer, the unit was assigned the maximum population density estimate from across all units to indicate there is no space available for mangroves to migrate landward. Units were then classified as ‘high’, ‘medium’ or ‘low’ coastal squeeze using terciles on logged values above 0, or as ‘none’ if population density was 0. If the future coastal development in a unit was projected to be lower than historical (i.e., a unit classified as ‘medium’ coastal development was projected to have ‘low’ coastal development), it was set to the historical category, assuming that any coastal development limiting landward migration of mangroves historically will not be removed in the future. | Gridded population projections for the coastal zone under the Shared Socioeconomic Pathways; https://figshare.com/s/9a94ae958d6a45684382 | Classified as high, medium or low coastal squeeze using terciles, or as ‘none’ if population density was 0. Coastal development was defined as present if population density was not ‘none’. |
| Historical pressure | Drought | Drought impacting mangroves. | Historical drought conditions were estimated for each mangrove forest unit from 1996-2020 using the Standardized Precipitation-Evapotranspiration index (SPEI; raster with 0.5° resolution; 12-month time-scale) ​(Beguria et al., 2022)​. The SPEI index is a standardised variable where values greater or less than 0 indicate anomalies from the mean, and negative values indicate drought conditions. Raster pixels with monthly SPEI values for each year between 1996 and 2020 were averaged within each unit to obtain average monthly SPEI values. Units that did not directly intersect with the SPEI raster were buffered by 10 kilometers to obtain average monthly SPEI values. All other units received a value of 0 (i.e., mean conditions). Drought was considered present between 1996 and 2020 if the minimum SPEI value for a unit was less than a percentile threshold of minimum SPEI values across all units. | SPEI index (Standardized Precipitation-Evapotranspiration); https://spei.csic.es/ | Present if drought anomaly was above a percentile threshold: 30^th^ (very lenient), 40^th^ (lenient), 50^th^ (moderate), 60^th^ (strict), and 70^th^ (very strict). |
| Future pressure | Drought | Future drought impacting mangroves. | Medium term (2041-2060) projections of percent change in the Standardised Precipitation Index (SPI) relative to baseline (1995-2014) conditions for each mangrove forest unit was estimated from an ensemble mean of CMIP6 projections under scenario SSP5-8.5 (raster with 1° resolution) ​(Gutierrez et al., 2021)​. The average value of raster pixels intersecting with each unit was calculated, and negative values represented potential future drought conditions. Units with a negative SPI percent change value less than a percentile threshold were considered at risk from drought. | IPCC WGI Interactive Atlas;  https://interactive-atlas.ipcc.ch | Present if projected negative percent change in SPI values was below a percentile threshold: 30^th^ (very lenient), 40^th^ (lenient), 50^th^ (moderate), 60^th^ (strict), and 70^th^ (very strict). |
| Historical pressure | Extreme high rainfall | Extreme rainfall impacting mangroves. | Extreme rainfall conditions were estimated for each mangrove forest unit from 1996-2020 using the Standardized Precipitation-Evapotranspiration index (SPEI; raster with 0.5° resolution; 12-month timescale) ​(Beguria et al., 2022)​. The SPEI index is a standardised variable where values greater or less than 0 indicate anomalies from the mean. We considered positive values to indicate extreme rainfall. Raster pixels with monthly SPEI values for each year between 1996 and 2020 were averaged within each unit to obtain average monthly SPEI values. Units that did not directly intersect with the SPEI raster were buffered by 10 kilometers to obtain average monthly SPEI values. All other units received a value of 0 (i.e., mean conditions). Extreme rainfall was considered present between 1996 and 2020 if the maximum SPEI value for a unit was above a percentile threshold of maximum SPEI values across all units. | SPEI index (Standardized Precipitation-Evapotranspiration); https://spei.csic.es/ | Present if extreme rainfall anomaly was above a percentile threshold: 30^th^ (very lenient), 40^th^ (lenient), 50^th^ (moderate), 60^th^ (strict), and 70^th^ (very strict). |
| Future pressure | Extreme high rainfall | Future extreme rainfall impacting mangroves. | Medium term (2041-2060) projections of estimated percent change in the Standardised Precipitation Index (SPI) relative to baseline (1995-2014) conditions for each mangrove forest unit was estimated from an ensemble mean of CMIP6 projections under scenario SSP5-8.5 (raster with 1° resolution) ​(Gutierrez et al., 2021)​. The average value of raster pixels intersecting with each unit was calculated, and positive values indicated potential future extreme rainfall. Units with a positive SPI percent change value greater than a percentile threshold were considered at risk from extreme rainfall. | IPCC WGI Interactive Atlas;  https://interactive-atlas.ipcc.ch | Present if projected positive percent change in SPI values was greater than a percentile threshold: 30^th^ (very lenient), 40^th^ (lenient), 50^th^ (moderate), 60^th^ (strict), and 70^th^ (very strict). |
| Historical pressure | Intense storms | Tropical storms impacting mangroves. | The number of tropical cyclones within a 200 km radius buffer from the centroid of each mangrove forest unit was calculated between 1996 and 2020 using the IBTrACS database ​(Knapp et al., 2010)​. As noted by ​(Hagger et al., 2022)​, a 200 km radius buffer represents the distance from a tropical cyclone’s eye within which a mangrove forest is likely to experience damage ​(Holland et al., 2010)​. Damage from intense tropical storms was considered present if the number of tropical cyclones intersecting a unit’s buffer was greater than a percentile threshold. | Tropical cyclone best track data (IBTrACS); https://climatedataguide.ucar.edu/climate-data/ibtracs-tropical-cyclone-best-track-data | Present if number of cyclones was above a percentile threshold: 30^th^ (very lenient), 40^th^ (lenient), 50^th^ (moderate), 60^th^ (strict), and 70^th^ (very strict). |
| Future pressure | Intense storms | The probability of a future intense tropical storm directly impacting mangroves by the year 2050. | The annual frequency of intense tropical storms under future climate conditions within a 200 km radius of the centroid of each mangrove forest unit was calculated. As noted by ​(Hagger et al., 2022)​, a 200 km radius buffer represents the distance from a tropical cyclone’s eye within which a mangrove forest is likely to experience damage ​(Holland et al., 2010)​. Annual tropical storm frequency was obtained by estimating the median number of synthetic tropical cyclone tracks projected per year for 10000 years under four different climate models from the HighResMIP (CMCC, CNRM, ECEARTH and HADGEM) at a resolution of 10 metres under scenario SSP5-8.5 (2015-2050) ​(Bloemendaal et al., 2022)​. The annual frequency of future cyclones (*f_cyc_*) was used to calculate the probability of a cyclone damaging a unit by the year 2050 with the following equation: p_cyc_ = 1 – (1 - *f_cyc_*)^^(2050-2023)^. Damage from future intense tropical storms was considered present if probability of cyclone occurring by 2050 was greater than a percentile threshold. | A globally consistent local-scale assessment of tropical cyclone risk; https://www.science.org/doi/10.1126/sciadv.abm8438 | Present if probability of cyclone occurring by 2050 was above a percentile threshold: 30^th^ (very lenient), 40^th^ (lenient), 50^th^ (moderate), 60^th^ (strict), and 70^th^ (very strict). |
| Historical pressure | Subsidence | Potential land subsidence from groundwater extraction impacting mangroves. | The probability of subsidence from groundwater extraction in 2010 was estimated for each mangrove forest unit from a raster (1km resolution) with pixels categorised according to 6 probability categories: 1 = very low, 2 = low, 3 = medium-low, 4 = medium-high, 5 = high, and 6 = very high ​(Herrera-Garcia et al., 2021)​. The mode of raster pixel values intersecting each unit was obtained. Units that did not directly intersect with the raster were buffered by 10 kilometres to obtain mode probability of subsidence. All remaining units without a value for subsidence probability were assigned 1 for ‘very low’ probability of subsidence. Subsidence was considered present in a unit if its probability was greater than a probability interval threshold. | Potential global land subsidence from groundwater extraction; https://figshare.com/articles/dataset/Global_Subsidence_Maps/13312070/1 | Present if probability of global subsidence was ≥ a probability interval: low (very lenient), medium low (lenient), medium high (moderate), high (strict), very high (very strict). |
| Future pressure | Subsidence | Future land subsidence from groundwater extraction impacting mangroves. | The probability of subsidence from groundwater extraction by 2040 was estimated for each mangrove forest unit. Probability of subsidence by 2040 was provided in raster pixels (1km resolution) categorised as: 1 = very low, 2 = low, 3 = medium-low, 4 = medium-high, 5 = high, and 6 = very high ​(Herrera-Garcia et al., 2021)​. The mode of all raster pixel values was obtained for each unit to represent the probability of subsidence by 2040. Units that did not directly intersect with the raster were buffered by 10 kilometres to obtain mode probability of subsidence. All remaining units without a value for subsidence probability were assigned 1 for ‘very low’ probability of subsidence. Subsidence was considered present in a unit if its probability was greater than a probability interval threshold. | Potential global subsidence from groundwater extraction by 2040; https://figshare.com/articles/dataset/Global_Subsidence_Maps/13312070/1 | Present if probability of global subsidence was ≥ a probability interval: low (very lenient), medium low (lenient), medium high (moderate), high (strict), very high (very strict). |
| Future pressure | Dams | Dams planned or under construction near river networks upstream of mangroves. | River networks from the HydroRivers database ​(Lehner & Grill, 2013)​ that drain into mangrove forest units were identified and buffered by 10 kilometres. The number of dams either planned or under construction in the future ​(Zarfl et al., 2015)​ that intersected with each unit’s buffered river network was calculated and converted to future dam presence-absence. Mangrove units without river networks received a value of 0. If Dams were present, sediment supply was considered to be low. | Future Hydropower Reservoirs and Dams (FHReD); https://www.globaldamwatch.org/fhred | Future dams present or absent. (No threshold used to define presence). |
| Biophysical context | Sediment supply | Proportion of sediment available in the catchment that is delivered to mangroves. | Catchments representing all land and river networks associated with a mangrove forest unit were created by identifying basins from the HydroSHEDS database that either directly intersect with a unit or are associated with rivers upstream of a unit ​(Linke et al., 2019)​. Basins associated with each unit were then dissolved to create a single catchment for each mangrove forest unit. The free-flowing river network (FFRN)​(Grill et al., 2019)​ was intersected with each mangrove catchment, and the coastal outlets of each unique river network was identified. The sediment trapping index provided in the FFRN represents the proportion of sediment trapped upstream in the catchment by anthropogenic barriers and was used as an estimate of land-based sediment supply to each unit. Where multiple rivers intersected with a unit, an average of the sediment trapping index values was taken, weighted by the rivers average long-term (1971-2000) naturalised discharge (m^3^s^-1^). Where the FFRN did not intersect with a mangrove catchment, the unit was given a value of 0, indicating sediment is not trapped by anthropogenic barriers in that catchment. Processing followed steps taken by Worthington et al. (under review) and Hagger et al. ​(Hagger et al., 2022)​. All units other than deltaic (i.e., lagoonal, estuarine and open coast) were considered to have low sediment supply. Deltaic units with a sediment trapping index greater than a percentage threshold were classified as having low sediment supply; if it was less than the percentage threshold the deltaic unit was classified as having high sediment supply. All non-deltaic units were classified as having low sediment supply. | Free-flowing River Network database; https://figshare.com/articles/dataset/Mapping_the_world_s_free-flowing_rivers_data_set_and_technical_documentation/7688801 | Deltaic units with a sediment trapping index greater than a percentage threshold were classified as having low sediment supply; if it was less than the percentage threshold the deltaic unit was classified as having high sediment supply. Units other than deltaic were considered to have low sediment supply. The percentage thresholds were: 30% (very lenient), 40% (lenient), 50% (moderate), 60% (strict), 70% (very strict). |
| Biophysical context | Tidal range | Tidal range; macro- (>4m), meso- (>2-4m) or microtidal (0-2m). | Tidal range was estimated for each mangrove forest unit using principal lunar semidiurnal or M2 tidal amplitude from the Finite Element Solution global tide model (FES2014; raster with 1/16° resolution) ​(Carrere et al., 2015)​. Units were split into individual forest patches, and each patch was assigned a tidal amplitude value from the raster pixel nearest to the centroid of the patch (with smallest value set 0.01m). Tidal amplitude for each unit was calculated as the mean of patch values, weighted by patch area relative to total unit area. Tidal range was estimated as the amplitude multiplied by 2, and units were classified as microtidal (0-2m), mesotidal (>2-4m) and macrotidal (>4m). Processing was the same as in Worthington et al. (under review). | Finite Element Solution tide model, FES2014; https://www.aviso.altimetry.fr | Classified as macro- (>4m), meso- (>2-4m) or microtidal (0-2m). |
| Biophysical context | Ecological connectivity | Average distance between extant mangrove forest and areas of historical loss. | Ecological connectivity in each mangrove forest unit was measured as the average minimum Euclidean distance (m) between the edges of extant mangrove forest patches in the year 2020 and the edges of patches lost between 1996 and 2019 ​(Bunting et al., 2022)​, standardised by total area of the unit (ha). In units where there was either no historical loss from 1996-2019 or no extant mangroves in 2020, the unit was assigned the maximum average distance calculated across all units. Units were classified as having ‘high’, ‘medium’, or ‘low’ establishment capacity using terciles, where large average distance values corresponded to low establishment capacity and vice versa. | Global Mangrove Watch; https://zenodo.org/record/6894273#.ZEXlTuxBxuU | Classified as ‘high’ if in the top tricile, ‘medium’ if in the middle tricile, and ‘low’ if in the bottom tercile. |
| Biophysical context | Aridity | Mangrove forest climatic conditions classified as either ‘arid’ or ‘humid. | Mangrove forests were classified as ‘arid’ or ‘humid’ according to aridity index values representative of average annual conditions between 1970-2000 ​(Zomer et al., 2022)​. The mean of all aridity index raster pixel values (1km resolution) intersecting each mangrove forest unit was taken to obtain a single value for each unit. For units that did not directly intersect the aridity raster, a 50-kilometre buffer was used to obtain a mean aridity index value. Following a generalised climate classification scheme (UNEP 1997), we classified all units with a mean aridity index value less than or equal to 0.5 as ‘arid’, and those greater than 0.5 as ‘humid’. | Global Aridity Index and Potential  Evapotranspiration (ET0) Database; Zomer and Trabucco et al. 2022; <https://cgiarcsi.community/2019/01/24/globalaridity->  index-and-potential-evapotranspiration-climate-database-v3/ | Classified as ‘arid’ if aridity index value was equal to or below 0.5, and ‘humid’ if above 0.5. |
| Hind-cast validation | Mangrove forest net loss and gain | Net loss or gain of mangrove forest from 1996-2020, classified as either seaward or landward. | Patches of mangrove loss or gain occurring throughout the Global Mangrove Watch 1996-2020 time series ​(Bunting et al., 2022)​ were intersected with boundaries representing oceanic exclusive economic zones ​(Flanders Marine Institute, 2019)​ to classify patches of loss or gain as ‘seaward’. Remaining areas of loss or gain were classified as ‘landward’. Total area of seaward and landward mangrove loss and gain from 1996-2020 was calculated in each mangrove forest unit, and net change was calculated by subtracting total losses from total gains. | Global Mangrove Watch; <https://zenodo.org/record/6894273#.ZEXlTuxBxuU>    Flanders Marine Institute; https://www.marineregions.org/. https://doi.org/10.14284/386 | Classified as either seaward or landward net loss or gain/stability. |

**References**

Belliard, J., Gourgue, O., Govers, G., Kirwan, M. L., & Temmerman, S. (2023). Coastal wetland adaptability to sea level rise: The neglected role of semi‐diurnal vs. diurnal tides. *Limnology and Oceanography Letters*. https://doi.org/10.1002/lol2.10298

Bunting, P., Rosenqvist, A., Hilarides, L., Lucas, R. M., Thomas, N., Tadono, T., Worthington, T. A., Spalding, M., Murray, N. J., & Rebelo, L.-M. (2022). Global Mangrove Extent Change 1996–2020: Global Mangrove Watch Version 3.0. *Remote Sensing*, *14*(15), 3657. https://doi.org/10.3390/rs14153657

Rogers, K. (2021). Accommodation space as a framework for assessing the response of mangroves to relative sea-level rise. *Singapore Journal of Tropical Geography*, *42*(2), 163–183. https://doi.org/10.1111/sjtg.12357

Worthington, T. A., zu Ermgassen, P. S. E., Friess, D. A., Krauss, K. W., Lovelock, C. E., Thorley, J., Tingey, R., Woodroffe, C. D., Bunting, P., Cormier, N., Lagomasino, D., Lucas, R., Murray, N. J., Sutherland, W. J., & Spalding, M. (2020). A global biophysical typology of mangroves and its relevance for ecosystem structure and deforestation. *Scientific Reports*, *10*(1), 14652. https://doi.org/10.1038/s41598-020-71194-5
